# Cationic Metal‐Organic Layer Delivers siRNAs to Overcome Radioresistance and Potentiate Cancer Radiotherapy

**DOI:** 10.1002/anie.202419409

**Published:** 2024-11-21

**Authors:** Xin Ma, Xiaomin Jiang, Zitong Wang, Yingjie Fan, Jinhong Li, Cathleen Chow, Chaoyu Wang, Chenghua Deng, Wenbin Lin

**Affiliations:** ^1^ Department of Chemistry The University of Chicago Chicago IL 60637 USA; ^2^ Department of Radiation and Cellular Oncology Ludwig Center for Metastasis Research The University of Chicago Chicago IL 60637 USA

**Keywords:** Metal-organic layers, Radiosensitizers, radiotherapy, Radioresistance, siRNA Delivery

## Abstract

Radiotherapy plays an important role in modern oncology, but its treatment efficacy is limited by the radioresistance of tumor cells. As a member of the inhibitor of apoptosis protein family, survivin plays a key role in developing radioresistance by mediating apoptosis evasion, promoting epithelial‐mesenchymal transition, and modulating cell cycle dynamics. Efficient downregulation of survivin expression presents a promising strategy to enhance the antitumor effects of radiotherapy. Herein, we report the design of a hafnium‐porphyrin‐based cationic metal‐organic layer (CMOL) with quaternary ammonium capping groups to deliver small interfering RNAs (siRNAs) for enhanced radiotherapy. The CMOL@siRNA nanoplatform not only increased energy deposition from X‐rays and reactive oxygen species generation via a unique radiotherapy‐radiodynamic therapy process, but also effectively delivered siRNAs to downregulate survivin expression and ameliorate radioresistance of cancer cells. Consequently, CMOL@siRNA in combination with low‐dose X‐ray irradiation demonstrated remarkable antitumor efficacy with 96.9 % and 91.4 % tumor growth inhibition in murine colorectal carcinoma and triple‐negative breast cancer models, respectively.

## Graphical abstract

A hafnium‐porphyrin‐based cationic metal‐organic layer (CMOL) delivers small interfering RNAs (siRNAs) to enhance radiotherapy. CMOL@siRNAs not only increases energy deposition from X‐rays and reactive oxygen species generation, but also effectively delivers an siRNA cocktail to downregulate survivin expression and ameliorate radioresistance of cancer cells. CMOL@siRNAs plus X‐ray irradiation shows remarkable antitumor efficacy in murine colorectal carcinoma and triple‐negative breast cancer models.

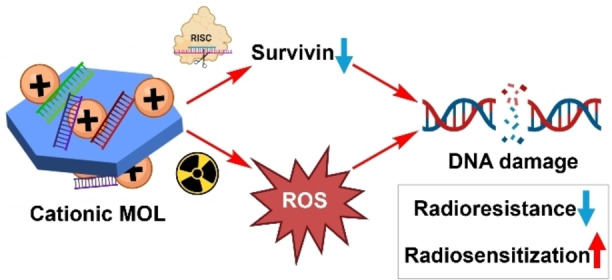

## Introduction

Radiotherapy (RT) harnesses the destructive power of ionizing radiation to control and eradicate tumors.[^1^, ^2^, ^3^] By inducing lethal DNA damage through generating DNA double‐strand breaks (DSBs) and oxidative stress in tumor cells,[^4^, ^5^] RT causes apoptosis of tumor cells to halt cancer progression and is used to treat more than half of all cancer patients. Despite its clinical utility, the radioresistance of evolving cancer populations diminishes the treatment efficacy of RT, leading to treatment failure, cancer recurrence, and poor patient outcomes.[^6^, ^7^]

One approach to enhance RT efficacy involves the use of radiosensitizers to sensitize tumor cells to radiation by increasing the energy deposition from ionizing radiation and the generation of reactive oxygen species (ROS).^[8]^ Recent advancements in nanotechnology have identified nanoradiosensitizers based on high‐Z element solid nanoparticles[^9^, ^10^, ^11^, ^12^] and nanoscale metal–organic frameworks (MOFs).[^13^, ^14^, ^15^, ^16^, ^17^, ^18^, ^19^] In particular, nanoscale MOFs have shown high radio‐enhancement effects via a unique radiotherapy‐radiodynamic therapy mechanism.[^20^, ^21^, ^22^, ^23^, ^24^] However, these nanoradiosensitizers do not address radioresistance of tumor cells, which compromises the therapeutic efficacy of nanoradiosensitizer‐mediated RT.

Extensive radiobiology studies have identified molecular pathways responsible for the radioresistance of tumor cells. For example, as a member of the inhibitor of apoptosis protein family, survivin suppresses caspase activation upon X‐ray irradiation of cancer cells, which enables them to evade apoptotic cell death.[^25^, ^26^, ^27^, ^28^] Survivin also promotes cell cycle progression to avoid G2/M arrest and facilitates DNA repair to scavenge radiation‐induced DSBs.^[29]^ Additionally, survivin affects cancer progression through its involvement in the epithelial‐mesenchymal transition (EMT), a critical process in cancer metastasis and therapy resistance. EMT is orchestrated by complex signaling networks involving transforming growth factor‐β (TGF‐β) and hypoxia‐inducible factor 1‐alpha (HIF‐1α), both of which regulate survivin expression[^30^, ^31^] and contribute to tumor aggressiveness under hypoxic conditions.[^32^, ^33^, ^34^] TGF‐β promotes EMT by downregulating epithelial markers and upregulating mesenchymal markers, facilitating tumor cell invasion and metastasis. HIF‐1α, activated in hypoxic tumors, regulates survivin expression through hypoxia‐responsive elements in its promoter region, enhancing cancer cell transcription after irradiation. Thus, the synergistic interactions of survivin, HIF‐1α, and TGF‐β allow cancer cells to evade apoptosis and promote metabolic adaptations to sustain tumor growth and progression.

We hypothesized that nanoradiosensitizers with desired surface characteristics could be used to deliver small interfering RNA (siRNA) cocktails targeting survivin, HIF‐1α, and TGF‐β to improve RT efficacy by increasing radiosensitivity of cancer cells. As double‐stranded RNAs, siRNAs bind to mRNAs via RNA‐induced silencing complexes (RISC), leading to mRNA degradation to silence the genes.[^35^, ^36^] Despite the power of siRNAs as a research tool in vitro, few delivery systems can protect siRNAs from rapid clearance and degradation to allow efficient gene silencing in vivo.

**Figure 1:**
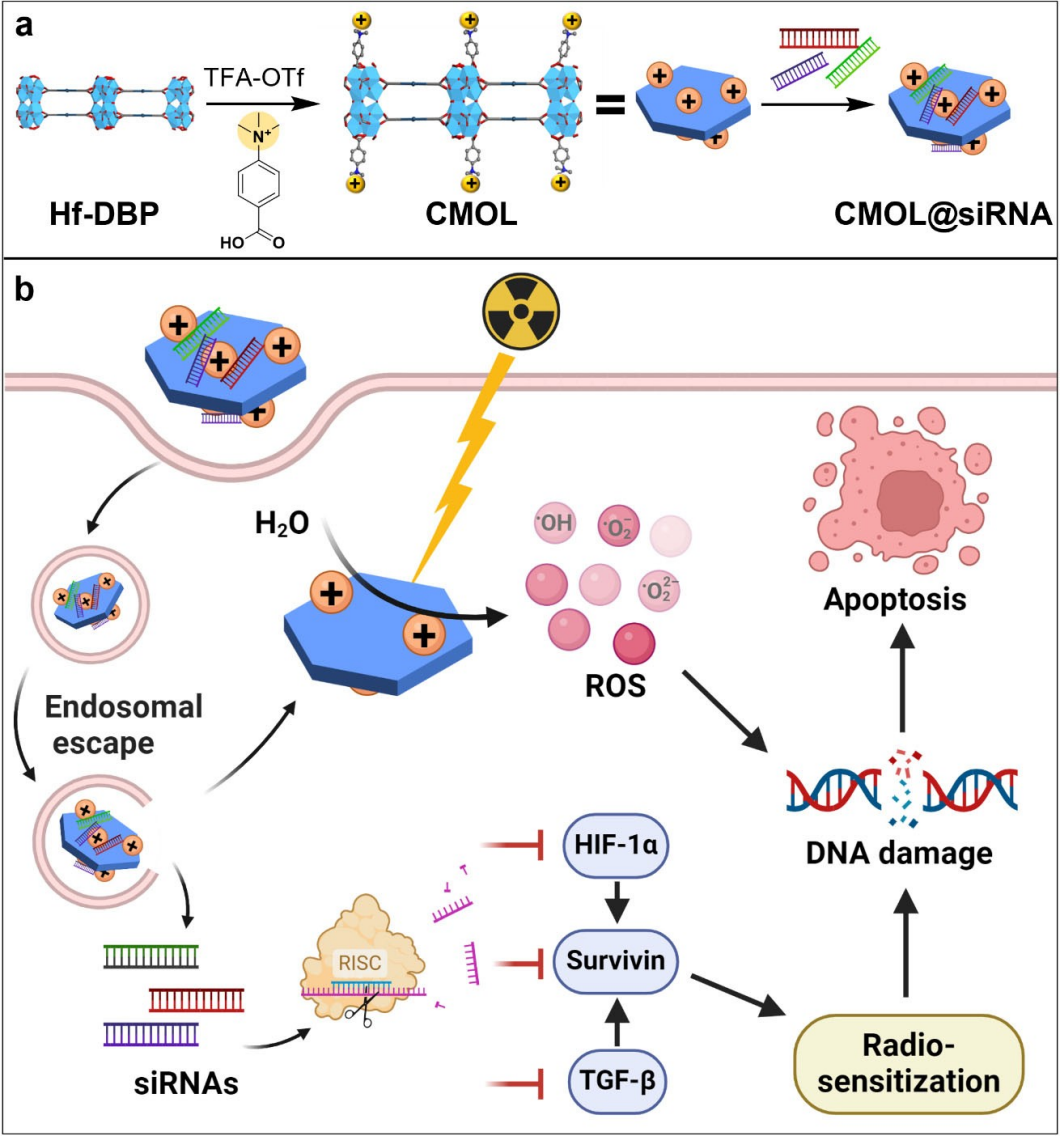
Schematic illustration of (a) synthesis and structure of CMOL@siRNAs and (b) siRNA delivery to increase the radiosensitivity of cancer cells. Created with BioRender.com.

Herein, we report the design of a radiosensitizing hafnium‐based cationic metal‐organic layer (CMOL) for the in vivo delivery of survivin‐targeted siRNA cocktails (CMOL@siRNAs) to increase radiosensitivity and enhance RT efficacy. The high‐Z metal‐based CMOL serves as an effective ROS generator, while the cationic framework functions as an efficient siRNA carrier to silence the expression of survivin, TGF‐β, and HIF‐1α to optimally downregulate survivin levels (Figure 1). CMOL@siRNAs augment tumor radiosensitivity during RT to enhance treatment efficacy with reduced side effects.

## Results and Discussion

### CMOL synthesis and siRNA loading

Hf‐DBP MOL was synthesized by a solvothermal reaction between HfCl_4_ and 5,15‐di(p‐benzoato) porphyrin (H_2_DBP) in *N,N‐*dimethylformamide (DMF) with propionic acid as a modulator, and then treated with trimethylsilyl trifluoroacetate to afford Hf‐MOL with labile trifluoroacetate (TFA) capping groups. Treatment of Hf‐MOL with 4‐carboxy‐*N,N,N*‐trimethylbenzenaminium chloride (TMBA) afforded cationic Hf‐MOL (CMOL) with TMBA capping agents.

**Figure 2:**
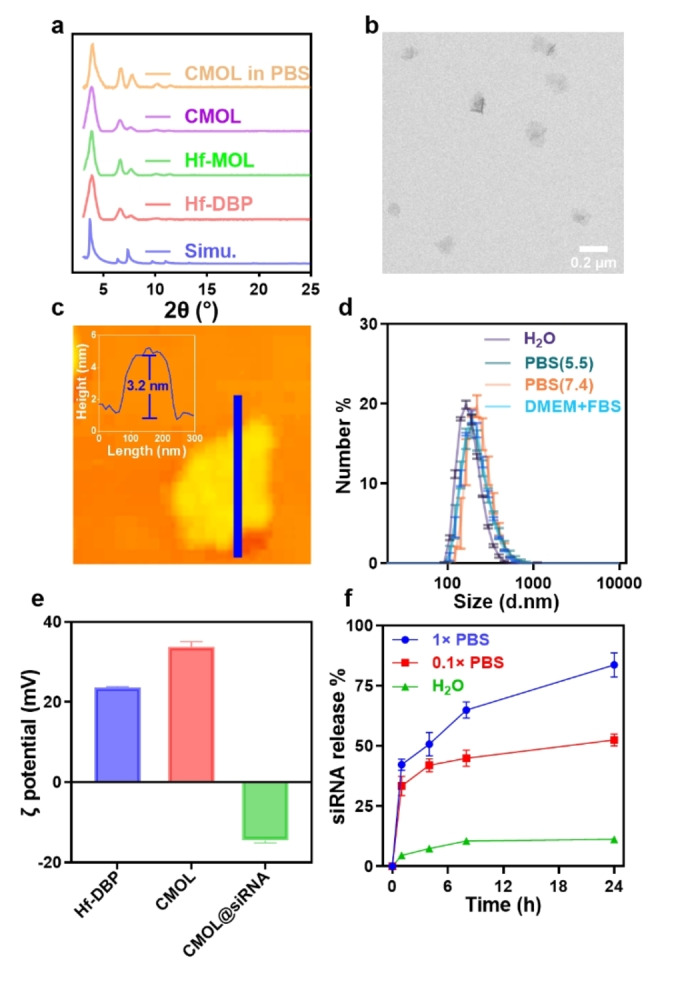
(a) PXRD patterns of Hf‐DBP, Hf‐MOL, and CMOL. (b) TEM and (c) AFM images of CMOL. (d) Number‐average size distribution of CMOL in H_2_O, PBS (pH 7.4), PBS (pH 5.5), and DMEM+10 % FBS (*n*=3). (e) Zeta potentials of Hf‐DBP, CMOL, and CMOL@siRNA. (f) siRNA release profiles in H_2_O, 0.1× PBS and 1×PBS (*n*=3). A scrambled sequence of siRNA, sc‐37007 (20–25 bp), was used in the characterization of CMOL@siRNA and as a negative control.

^1^H NMR analysis of the digested CMOL indicated successful loading of cationic TMBA groups on Hf_12_ SBUs with a TMBA/DBP ratio of 0.34 (Figure S1–4). Powder X‐ray diffraction (PXRD) and transmission electron microscopy (TEM) demonstrated that the crystallinity and morphology of Hf‐DBP MOL were retained throughout the post‐synthetic modification processes (Figure 2a, 2b, S5). Atomic force microscopy (AFM, Figure 2c) imaging indicated the single‐layer crystalline nanosheet morphology for the MOLs. CMOL showed a thickness of ~3.2 nm, which matches the height of the simulated TMBA‐capped Hf_12_‐SBU (3.0 nm, Figure S6). Hf‐DBP showed a size of 178.9 nm with a polydispersity index (PDI) of 0.108 by dynamic light scattering (DLS). After post‐synthetic modifications, CMOL showed a similar size of 180.3 nm and a PDI of 0.129 (Figure S7). CMOL maintained its crystallinity in phosphate‐buffered saline (PBS) (Figure 2a) and exhibited colloidal stability in PBS and cell culture media with unchanged sizes (Figure 2d). CMOL also exhibited a more positive surface charge than Hf‐DBP due to surface modification with TMBA groups (Figure 2e).

Incubation of CMOL with a control siRNA for 30 minutes followed by centrifugation afforded CMOL@siRNA. The loading efficiency of siRNA on CMOL reached 100 % when the weight ratio of CMOL to siRNA was larger than 5 (Table S1). The surface charge was reversed after siRNA loading (Figure 2e). CMOL@siRNA showed only ~10 % release of siRNA after incubation in water for 8 hours but released ~45 % siRNA in 0.1× PBS and ~65 % siRNA in 1× PBS, respectively, in 8 hours (Figure 2f), suggesting a phosphate‐dependent release kinetics that can facilitate intracellular release of siRNA from CMOL@siRNA owing to the higher phosphate concentration inside cells.

### Intracellular uptake and siRNA transfection of CMOL@siRNAs

Flow cytometric analysis supported intracellular delivery of fluorescein isothiocyanate (FITC)‐labeled siRNA (siRNA^FITC^) by CMOL in 4T1 murine triple‐negative breast cancer cells (Figure S8). CMOL@siRNA^FITC^ treatment significantly increased the FITC signal over free siRNA^FITC^ treatment, indicating enhanced siRNA cellular uptake of CMOL@siRNA. The mean fluorescence intensity (MFI) of CMOL@siRNA^FITC^ reached the highest value of 992 at a CMOL concentration (based on Hf) of 30 μM. The control group with free siRNA^FITC^ treatment shows a significantly lower MFI of 561. CMOL@siRNA efficiently enhanced the cellular uptake of siRNA, with 99 % of uptake occurring even at the lowest CMOL concentration of 15 μM. The steadily decreased uptake percentages of 92.0 % and 81.5 % at higher CMOL concentrations of 60 μM and 100 μM suggest that the cellular uptake reaches a plateau due to saturation of the MOL uptake capacity of the cells. Thus, a CMOL concentration of 30 μM (weight ratio of CMOL/siRNA=10) was selected for subsequent studies.

**Figure 3:**
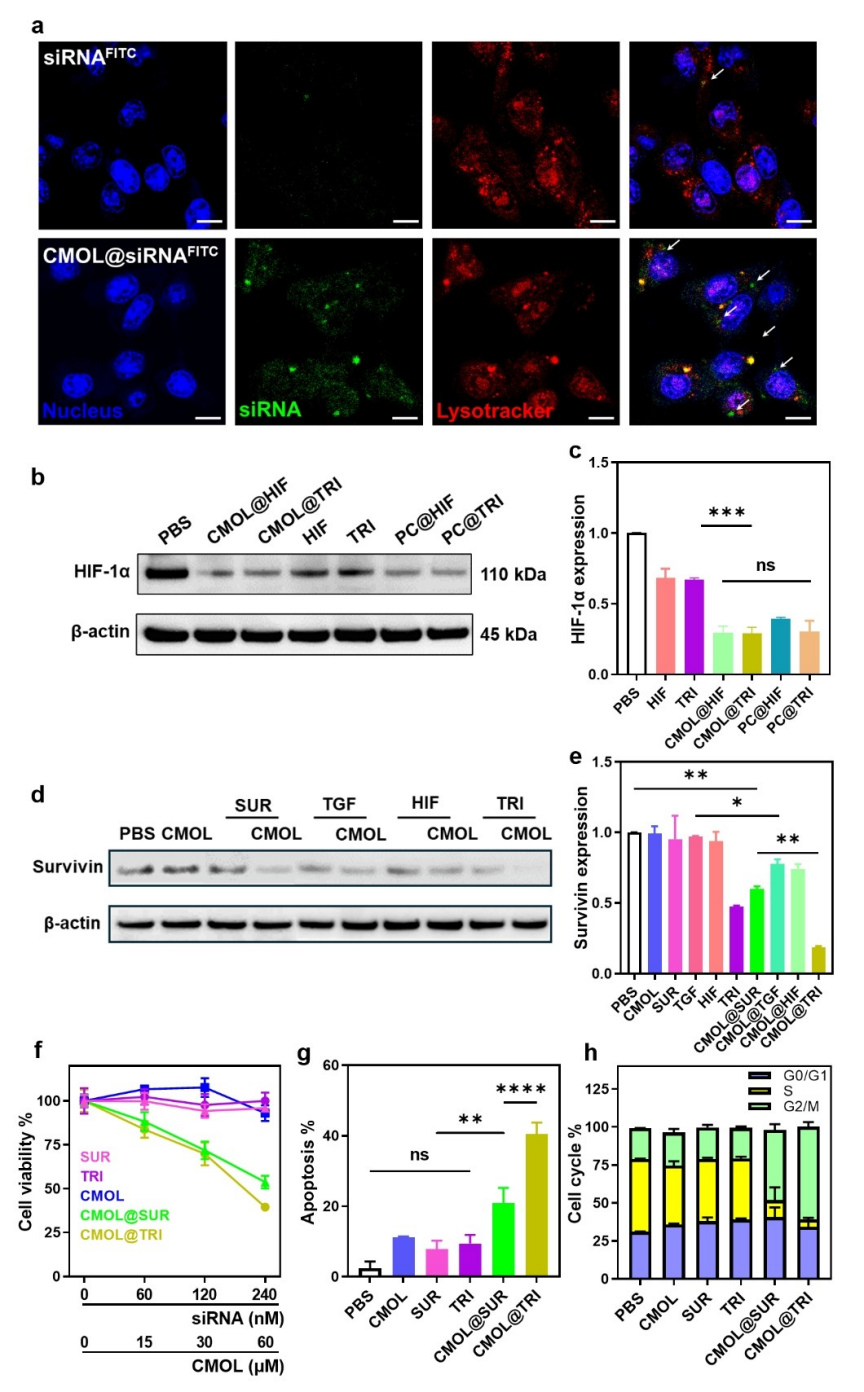
(a) CLSM imaging of intracellular distributions of siRNA^FITC^ and CMOL@siRNA^FITC^ in 4T1 cells. Nucleus was stained by Hoechst 33342 (blue), siRNA was labeled by FITC (green), and endo/lysosomes were stained by Lyso Tracker DND‐99 (red). (b) Relative HIF‐1α expression in 4T1 cells after different treatments. The corresponding uncropped images are included in Figure S42. (c) Western blot analysis of HIF‐1α expression in 4T1 cells after different treatments. (d) Relative survivin expression in 4T1 cells after different treatments. The corresponding uncropped images are included in Figure S43. (e) Western blot analysis of survivin expression in 4T1 cells after different treatments (*n*=3). (f) Viability curves (mean±SD %) of 4T1 cells after treatment with CMOL, SUR, TRI, CMOL@SUR, or CMOL@TRI. (*n*=3). (g) Apoptosis assay of 4T1 cells after different treatments (*n*=3). (h) Cell cycle assay of 4T1 cells after different treatments (*n*=3). *p<0.05, **p<0.01, ***p<0.001, ****p<0.0001.

Confocal laser scanning microscopy (CLSM) showed that 4T1 cells treated with free siRNA^FITC^ displayed minimal green fluorescence, consistent with inefficient siRNA internalization (Figure 3a). Weak colocalization between siRNA^FITC^ and LysoTracker (Pearson R value=0.46) was observed, suggesting poor intracellular trafficking, fast siRNA degradation, and limited endo/lysosomal escape.[^37^, ^38^] In contrast, CMOL@siRNA^FITC^ increased the green fluorescence by almost 10‐folds over free siRNA^FITC^, indicating a significantly higher uptake of CMOL@siRNA by 4T1 cells. Moreover, strong colocalization between siRNA^FITC^ and LysoTracker was observed in the spots with yellow signals in the merged image, suggesting internalization of CMOL@siRNA^FITC^ and its trafficking to endo/lysosomes. Interestingly, numerous green siRNA^FITC^ signals (indicated by the white arrows with Pearson R value as low as 0.18) were observed in the merged image, indicating efficient escape of CMOL@siRNA^FITC^ or siRNA^FITC^ from endo/lysosomes. These results show that CMOL@siRNA^FITC^ effectively delivers siRNA^FITC^ to 4T1 cells and enables endo/lysosomal escape of siRNA^FITC^.

We then loaded CMOL with a siRNA cocktail consisting of siRNA^survivin^ (SUR), siRNA^TGF−β^ (TGF), and siRNA^HIF−1α^ (HIF) to afford CMOL@TRI. We studied the transfection efficiency of CMOL@TRI and control groups by quantifying the expression of survivin, TGF‐β, and HIF‐1α proteins. The commercial siRNA transfection agent X‐tremeGENE 360 was used as a positive control (PC). Protein expression was normalized to β‐actin and PBS treatment served as a control with a relative expression of 1.

4T1 cells were treated with increasing concentrations of CoCl_2_ at 50, 100, 200, and 500 μM to induce HIF‐1α upregulation, which increased HIF‐1α expression by 18‐, 23‐, 30‐, and 350‐folds, respectively, over PBS control based on western blot analysis (Figure S9). However, 500 μM CoCl_2_ significantly reduced β‐actin expression due to its cytotoxicity. 200 μM CoCl_2_ was selected for subsequent HIF‐1α gene silencing studies. 4T1 or CT26 cells were treated with 200 μM CoCl_2_ in combination with various siRNA formulations for 24 hours to assess the impact on HIF‐1α expression.

In 4T1 cells, CMOL reduced HIF‐1α expression by 10.2 %, whereas PC downregulated HIF‐1α expression by 22.3 % (Figure 3b, 3c). Free HIF reduced HIF‐1α expression by 31.6 %. As expected, CMOL@HIF and PC@HIF significantly reduced HIF‐1α expression by 70.4 % and 60.6 %, respectively. Similarly, free TRI downregulated HIF‐1α expression by 32.9 %, while CMOL@TRI and PC@TRI reduced HIF‐1α expression by 70.7 % and 69.6 %, respectively. These results show that CMOL outperforms PC in delivering siRNAs for gene silencing. In CT26 cells, HIF and CMOL@HIF downregulated HIF‐1α expression by 18.6 % and 40.5 %, respectively, while TRI and CMOL@TRI downregulated HIF‐1α expression by 7.5 % and 48.3 %, respectively (Figure S10).

We also examined the silencing of TGF‐β in 4T1 cells. While CMOL slightly decreased TGF‐β expression from PBS control by 7.5 % (Figure S11), CMOL@TGF and CMOL@TRI significantly downregulated TGF‐β expression by 73.0 % and 81.9 %, respectively. CMOL@TGF and CMOL@TRI also effectively reduced TGF‐β expression in CT26 cells by 67.3 % and 74.0 %, respectively, relative to PBS control (Figure S12). In contrast, TGF and TRI failed to reduce TGF‐β expression in CT26 cells.

The impact of siRNA delivery on survivin expression was next examined. CMOL had no effect on survivin downregulation whereas SUR, TGF, and HIF slightly reduced survivin expression by 4.7 %, 2.7 % and 6.3 %, respectively (Figure 3d, 3e). CMOL@SUR significantly downregulated survivin expression by 40.0 %. Interestingly, CMOL@HIF and CMOL@TGF reduced survivin expression by 25.8 % or 22.1 %, respectively, indicating the dependence of survivin expression on multiple pathways and the potential of effective survivin downregulation using a cocktail of siRNAs. Indeed, with a siRNA cocktail targeting survivin, TGF‐β, and HIF‐1α, TRI efficiently reduced survivin expression by 52.3 %. CMOL@TRI efficiently downregulated survivin by 81.0 %. The siRNA delivery efficiency was preserved after storage of CMOL@TRI at 4 °C for one week (Figure S13). The mRNA levels of survivin and HIF‐1α after treatments were also assessed by real‐time quantitative polymerase chain reaction analysis in 4T1 cells. TRI and CMOL@TRI downregulated mRNA levels of HIF‐1α by 17.7 % and 88.5 %, respectively, and reduced survivin mRNA levels by 44.3 % and 86.5 %, respectively (Figure S14). These results establish CMOL as a robust nanoplatform for delivering siRNA cocktails for gene silencing and highlight the potential of efficiently downregulating survivin with CMOL@TRI by simultaneously targeting multiple signaling pathways.

### In vitro anticancer effects of CMOL@siRNAs

CMOL did not show cytotoxicity at a Hf concentration of 120 μM (Figure S15) and SUR and TRI at 240 nM were nontoxic to 4T1 and CT26 cells by MTS assay (Figure 3f, S16). The delivery of siRNAs by CMOL significantly increased their cytotoxicity. CMOL@SUR and CMOL@TRI showed siRNA half‐maximal inhibitory concentration (IC_50_) values of 308.2 and 219.7 nM, respectively, in 4T1 cells, and 185.5 and 131.8 nM, respectively, in CT26 cells. These results demonstrate the exceptional ability of CMOL in delivering siRNAs to cancer cells to induce cell death and the synergistic effects of siRNA cocktails targeting survivin genes.

Apoptosis assays by flow cytometry supported the MTS results. While SUR and TRI induced minimal apoptosis (7.8 % and 9.3 % in 4T1 cells and 8.5 % and 9.1 % on CT26 cells, respectively) due to their poor cellular uptake and gene silencing efficiency, CMOL@SUR and CMOL@TRI showed much higher apoptosis rates (20.9 % and 40.5 % in 4T1 cells and 16.6 % and 23.2 % in CT26 cells, respectively) (Figure 3g, S17, S18). These results further support the survivin downregulation strategy using CMOL‐delivered siRNA cocktails targeting survivin, TGF‐β, and HIF‐1α pathways.

Cell cycle analysis of CMOL@siRNA‐treated 4T1 and CT26 cells provided further evidence for efficient siRNA delivery and transfection by CMOL. PBS, CMOL, SUR and TRI treatments did not alter normal cell cycle distributions, with 30–35 % of cells in G0/G1 phase, 45–50 % in S phase, and 20–25 % in G2/M phase in 4T1 cells (Figure 3h, S19), and 53–55 % of cells in G0/G1 phase, 35–40 % in S phase, and 5–10 % in G2/M phase in CT26 cells (Figure S20). In contrast, CMOL@SUR and CMOL@TRI induced significant cell cycle arrest in the G2/M phase, which is a critical checkpoint for cell division and has the highest radiosensitivity.^[39]^ CMOL@SUR increased the G2/M phase proportion by 2.3‐folds to 46.3 % from 20.2 % for PBS control in 4T1 cells and by 2.9‐folds to 28.7 % from 9.9 % for PBS control in CT26 cells. CMOL@TRI increased the G2/M population by 3.0‐folds over PBS in 4T1 cells and by 4.5‐folds over PBS in CT26 cells. Thus, CMOL@TRI effectively disrupts cell cycle and causes G2/M arrest. As cancer cells in G2/M arrest are highly sensitive to radiation damage, we hypothesized that CMOL@TRI could be combined with radiotherapy to induce cell death and improve therapeutic outcomes.

### In vitro anticancer effects of CMOL@siRNAs plus X‐ray irradiation

**Figure 4:**
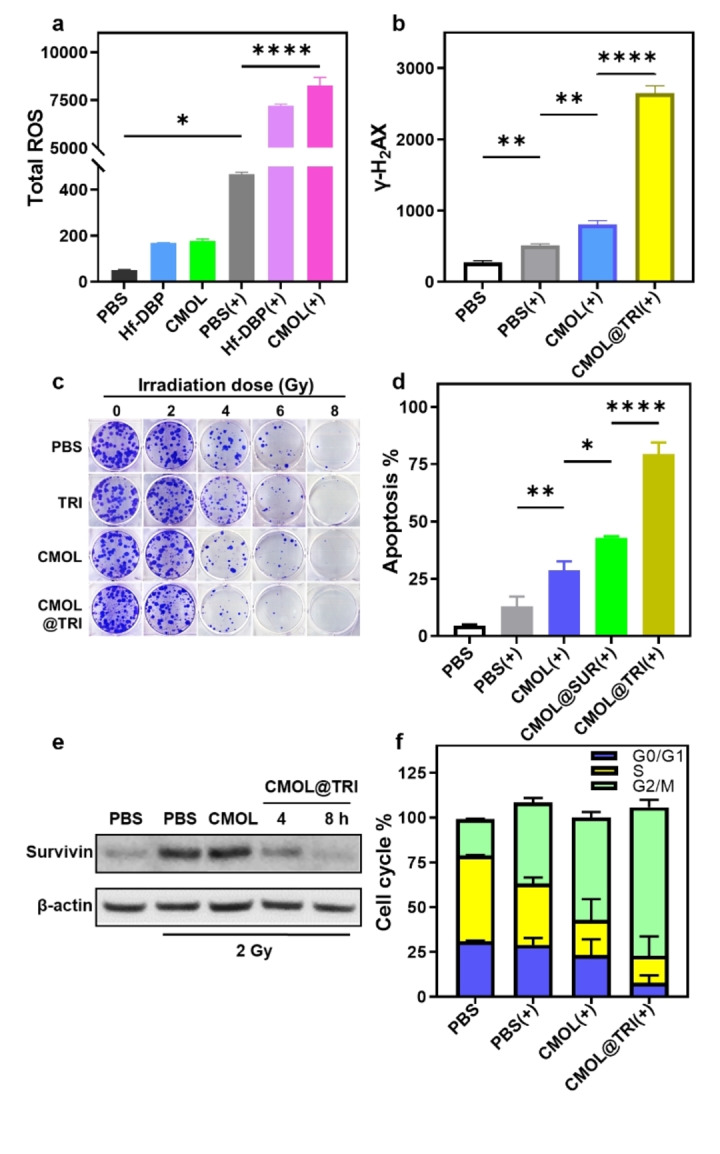
(a) Total ROS signals in 4T1 cells after 4 Gy X‐ray irradiation (*n*=3). (b) γ‐H2AX signals in 4T1 cells after 4 Gy X‐ray irradiation as determined by CLSM. (c) Representative images of 4T1 colonies 7 days after different treatments plus X‐ray irradiation (*n*=3). (d) Apoptosis assay of 4T1 cells after different treatments plus X‐ray irradiation (*n*=3). (e) Western blots of survivin expressions in 4T1 cells upon 2 Gy of X‐ray irradiation. The corresponding uncropped images are included in Figure S44. (f) Cell cycle analysis of 4T1 cells after different treatments plus X‐ray irradiation (*n*=3). *p<0.05, **p<0.01, ***p<0.001, ****p<0.0001.

Radiotherapy has been shown to upregulate survivin expression which induces radioresistance of tumor cells.^[40]^ CMOL plus 4 Gy X‐ray irradiation [denoted CMOL(+)] significantly increased reactive oxygen species (ROS) generation by 171.2‐folds over PBS, 50.9‐folds over CMOL, and 18.6‐folds over PBS(+) (Figure 4a, S21). The significant increase in ROS levels suggests that CMOL can enhance the radiotherapeutic effect of X‐ray irradiation by increasing energy deposition and oxidative stress in cancer cells.

As a biomarker for DNA DSBs,^[41]^ γ‐H_2_AX was significantly upregulated in 4T1 cells treated with CMOL(+) and CMOL@TRI(+) as shown by CLSM (Figure 4b, S22). Compared to PBS(+), CMOL(+) increased γ‐H_2_AX expression by 1.5‐folds while CMOL@TRI(+) increased γ‐H_2_AX expression by 5.2‐folds. These results indicated that TRI delivered by CMOL significantly increased X‐ray‐induced DNA damage in cancer cells.

Clonogenic assays showed X‐ray dose‐dependent inhibition of 4T1 cell colony formation by PBS, TRI, CMOL, and CMOL@TRI (Figures 4c, S23). CMOL@TRI, TRI, and CMOL showed radiation enhancement factor at 10 % survival (REF_10_) values of 1.5, 1.1, and 1.2, respectively (Figure S24). This result indicated that CMOL@TRI(+) significantly inhibited long‐term proliferation of 4T1 cells over other groups. Apoptosis assays supported these findings. At 6 Gy of X‐ray, CMOL@TRI(+) induced 80 % and 85 % apoptosis in 4T1 cells and CT26 cells, respectively (Figure 4d, S25, S26). In comparison, CMOL(+) induced 26.2 % apoptosis in 4T1 cells and 61.1 % apoptosis in CT26 cells, whereas CMOL@SUR(+) induced 43.4 % apoptosis in 4T1 cells and 69.8 % apoptosis in CT26 cells. The lower apoptosis rates in 4T1 cells than those in CT26 cells in PBS(+), CMOL(+), and CMOL@SUR(+) groups are likely caused by radiotherapy‐induced radioresistance in 4T1 cells.^[42]^ CMOL@TRI(+) likely increased cell killing by increasing radiosensitivity of 4T1 cells.

Western blot analysis indicated CMOL(+) slightly upregulated survivin by 12 % over PBS(+) (Figures 4e, S27). Interestingly, CMOL@TRI(+) effectively downregulated survivin expression by 46.3 % and 65.4 % at 4 and 8 hours, respectively over PBS(+). Thus, effective survivin downregulation by CMOL@TRI(+) increased radiosensitivity of 4T1 cells to promote cell death, likely by inhibiting DNA damage repair process which can be facilitated by survivin upregulation.^[43]^

Cell cycle analysis showed that CMOL@TRI(+) induced significant mitotic disruption in 4T1 and CT26 cells. For CT26 cells, PBS(+) slightly increased the cells in the G2/M phase to 25 % from 10 % for PBS, indicating that low‐dose X‐ray irradiation has a limited impact on cell cycle arrest (Figure S28). CMOL(+) increased the G2/M phase distribution to approximately 50 %, suggesting that CMOL enhances the response to radiotherapy by arresting cells at this critical checkpoint. CMOL@TRI(+) treatment accumulated 65 % CT26 cells in the G2/M phase, indicating the amplification of the radiotherapy effects by CMOL@TRI through cell cycle arrest to increase their susceptibility to radiation‐induced DNA damage. The mitotic disruption was more notable in 4T1 cells with higher radioresistance. PBS(+) slightly increased 4T1 cells in the G2/M phase to ~35 % from ~20 % for PBS (Figure 4f, S29). CMOL(+) and CMOL@TRI(+) further increased the proportions of 4T1 cells in the G2/M phase to approximately 55 % and 85 %, respectively. These results demonstrate CMOL@TRI as a potent radiosensitizing agent to enhance radiotherapy of resistant cancer cells.

### Intratumoral retention and in vivo antitumor efficacy of CMOL@siRNA

**Figure 5:**
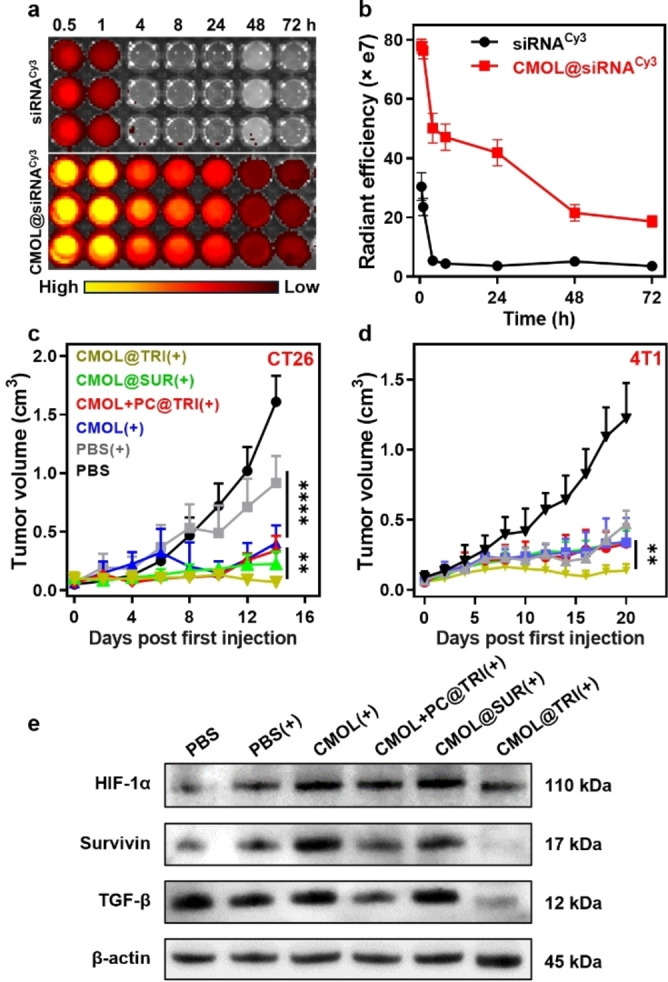
(a,b) Photos (a) and quantifications (b) of time‐dependent fluorescence signals of siRNA^cy3^ and CMOL@siRNA^cy3^ in CT26 tumor lysates (*n*=3). (c,d) Growth curves of subcutaneous CT26 tumors (c) and 4T1 tumors (d) after different treatments (*n*=5). (e) In vivo western blots analysis of HIF‐1α, survivin, TGF‐β, and expression on 4T1 tumors after different treatments. The corresponding uncropped images are included in Figure S45. *p<0.05, **p<0.01, ***p<0.001, ****p<0.0001.

The intratumoral retention of Cy3 fluorescently labeled siRNA (siRNA^cy3^) was assessed using the in vivo imaging system (IVIS) in CT26 tumor model. CMOL@siRNA^cy3^ extended siRNA^cy3^ half‐life from 0.4 h to 7.2 h and increased areas‐under‐curves (AUCs) by 6.8‐folds over free siRNA^cy3^ (Figure 5a, 5b). These results indicate that CMOL@siRNA provides a promising platform for siRNA delivery to increase radiosensitivity of tumors by silencing survivin genes in vivo.

We evaluated the antitumor efficacy of CMOL@TRI(+) in subcutaneous CT26 and 4T1 tumor models in BALB/c mice. When the tumors reached approximately 100 mm^3^ on day 7 post‐tumor cell inoculation, the mice were intratumorally injected with PBS, CMOL, CMOL plus siRNA delivered with PC (CMOL+PC@TRI), CMOL@SUR, CMOL@TGF, CMOL@HIF, TRI or CMOL@TRI twice on day 7 and 9. The tumors in selected groups were irradiated with 3 Gy X‐ray for 4 daily fractions on day 8 through 11 for CT26 model and for 6 daily fractions on day 8 through 13 for 4T1 model (Figure S30).

For CT26 tumor‐bearing mice, PBS(+) moderately slowed tumor growth with a tumor growth inhibition (TGI) value of 0.543 (Table S2, Figure 5c), reflecting limited antitumor effects of low‐dose radiotherapy. CMOL(+) showed a TGI of 0.819 due to the radiosensitizing effect of CMOL. Loading of CMOL with SUR, TGF, and HIF further enhanced the antitumor efficiency with TGI values of 0.931, 0.924, and 0.921 %, respectively (Figure S31). CMOL+PC@TRI(+) did not enhance tumor growth inhibition over CMOL(+) with a TGI of 0.813. In contrast, CMOL@TRI(+) potently regressed the tumors to afford the highest TGI of 0.969. All treatment groups showed steady body weights during the study (Figure S32). Thus, CMOL@TRI(+) provides a substantial improvement in therapeutic efficacy compared to all other treatments, likely due to the synergistic silencing of survivin genes by the siRNA cocktail.

Immunohistochemistry staining (IHC) studies showed that CMOL@TRI(+) drastically increased γ‐H_2_AX expression over CMOL(+) and CMOL+PC@TRI(+), which provide direct evidence for enhanced radiation‐induced DNA damage over CMOL(+) and CMOL+PC@TRI(+) (Figure 5e, S33). Hematoxylin and eosin (H&E) staining and terminal deoxynucleotidyl‐transferase nick‐end‐labeling (TUNEL) staining studies showed that CMOL@TRI(+)‐treated tumors exhibited lower nuclear densities and more DNA fragmentations over other treatment groups, indicating reduced cancer cell proliferation and enhanced apoptosis from CMOL@TRI(+) treatment. All treatment groups except CMOL+PC@TRI(+) showed no histology abnormality of normal organs, indicating the lack of general toxicity. In the CMOL+PC@TRI(+) group, the pink‐stained areas displayed loss of cellular details, increased interstitial cellularity, and tubular damages (Figure S34), suggesting moderate inflammation and cell death in the kidneys.^[44]^ This nephrotoxicity was likely caused by the cationic surfactant in PC.

The antitumor effect of CMOL@TRI(+) was confirmed in the 4T1 tumor model. While PBS(+) showed a modest TGI of 0.388, CMOL(+) and CMOL+PC@TRI(+) slightly improved the TGI values to 0.535 and 0.502, respectively (Figure 5f). CMOL@SUR(+) was only slightly more effective than CMOL(+) with a TGI of 0.582. In contrast, CMOL@TRI(+) drastically increased the TGI value to 0.914 without showing any sign of adverse effects (Figure S35). These results highlight the importance of silencing multiple signaling pathways with an siRNA cocktail to overcome radioresistance, and establish CMOL@TRI as a potent bifunctional radiosensitizer to overcome tumor radioresistance and enhance radiotherapeutic efficacy.

western blot analysis showed that PBS(+) and CMOL(+) slightly reduced TGF‐β expression to 0.90 and 0.86, respectively, from PBS (1.0) in 4T1 tumors (Figure 5d, S36a). CMOL@TRI(+) significantly downregulated TGF‐β expression to approximately 0.39 while CMOL+PC@TRI(+) reduced TGF‐β expression to 0.64. These results showed that CMOL@TRI(+) much more effectively downregulated TGF‐β expression than other groups. While PBS(+) slightly increased HIF‐1α expression to 1.55 over PBS (1.0), CMOL(+) and CMOL+PC@TRI(+) significantly upregulated HIF‐1α expression to 1.86 and 1.81, respectively (Figure S36b), indicating radiation‐induced HIF‐1α upregulation to temper the radiotherapeutic effect. Interestingly, CMOL@TRI(+) reversed radiation‐induced HIF‐1α upregulation to a similar level of 1.08 as PBS control.

While PBS(+) upregulated survivin expression to 1.50 from PBS (1.0), CMOL(+) further increased survivin expression to 2.26 (Figure S36c). This result indicated significant upregulation of survivin in response to radiation to cause radioresistance. CMOL+PC@TRI(+) and CMOL@SUR(+) slightly reduced survivin expression from CMOL(+) by 29.6 % and 20.3 % to 1.59 and 1.80, respectively. Importantly, CMOL@TRI(+) significantly downregulated survivin expression from CMOL(+) by 74.3 % to 0.58, indicating the ability to effectively silence survivin by the siRNA cocktail to overcome radioresistance.

## Conclusion

In this work, we developed a novel cationic MOL for siRNA delivery to enhance radiotherapy and overcome radioresistance. The postsynthetic modification of Hf‐DBP MOL with quaternary ammonium capping groups afforded CMOL with superior siRNA delivery and transfection efficiencies than a commercial lipid‐based siRNA carrier. CMOL@siRNAs in combination with low‐dose X‐rays induced significant DNA damage, disrupted mitosis by arresting cancer cells in the radiosensitive G2/M phase, and induced strong apoptosis in cancer cells. CMOL increased tumor retention of siRNAs in vivo by 6.8‐folds to efficiently downregulate survivin expression via silencing multiple signaling pathways with an siRNA cocktail. As a result, CMOL@siRNAs in combination with low‐dose X‐ray irradiation demonstrated remarkable antitumor efficacy with 96.9 % and 91.4 % tumor growth inhibition in murine colorectal carcinoma and triple‐negative breast cancer models, respectively. These findings underscore the potential of MOLs as a multifunctional nanoplatform for siRNA delivery to combine with other treatment modalities to overcome resistance and enhance therapeutic efficacy.

## Supporting Information

The authors have cited additional references within the Supporting Information.

## Conflict of Interests

The authors declare no conflict of interest.

1

## Supporting information

As a service to our authors and readers, this journal provides supporting information supplied by the authors. Such materials are peer reviewed and may be re‐organized for online delivery, but are not copy‐edited or typeset. Technical support issues arising from supporting information (other than missing files) should be addressed to the authors.

Supporting Information

## Data Availability

The data that support the findings of this study are available from the corresponding author upon reasonable request.
