# Supplementary material for: Cationic Metal‐Organic Layer Delivers siRNAs to Overcome Radioresistance and Potentiate Cancer Radiotherapy
Source: Angew Chem Int Ed Engl. 2024 Nov 21;64(7):e202419409. doi: 10.1002/anie.202419409 (PMC11811689; doi:10.1002/anie.202419409)
Supplement: Supplementary file 1 — Supporting Information [file ANIE-64-e202419409-s001.pdf]

Angewandte Chemie
Eine Zeitschrift der Gesellschaft Deutscher Chemiker

Supporting Information

Cationic Metal-Organic Layer Delivers siRNAs to Overcome Radioresistance and Potentiate Cancer Radiotherapy

*X. Ma, X. Jiang, Z. Wang, Y. Fan, J. Li, C. Chow, C. Wang, C. Deng, W. Lin**

Supporting Information for

Cationic Metal-Organic Layer Delivers siRNAs to Enhance Radiosensitivity and Potentiate Cancer Radiotherapy

Xin Ma,† [a] Xiaomin Jiang,† [a,b] Zitong Wang,† [a] Yingjie Fan,[a] Jinhong Li,[a] Cathleen Chow,[a] Chaoyu Wang,[a,b] Chenghua Deng,[a], Wenbin Lin*[a,b]

[a]Department of Chemistry, The University of Chicago, Chicago, Illinois 60637, United States

[b]Department of Radiation and Cellular Oncology and Ludwig Center for Metastasis Research, The University of Chicago, Chicago, Illinois 60637, United States

KEYWORDS: Metal-organic layers • Radiosensitizers • radiotherapy • Radioresistance • siRNA Delivery

S1 Experimental Section

S1.1 Materials and Methods

All starting materials were purchased from Sigma-Aldrich and ThermoFisher (USA) unless otherwise noted and used without further purification. Transmission electron microscopy (TEM) was carried out on a TECNAI Spirit and a TECNAI F30 HRTEM. Atomic force microscopy (AFM) images were taken on a Bruker Multimode 8-HR instrument. Powder X-ray diffraction (PXRD) data was collected on a Bruker D8 Venture diffractometer using a Cu Kα radiation source (λ = 1.54178 Å) and processed with PowderX software. UV-Vis spectra were collected using a Shimadzu UV-2600 UV-Vis spectrophotometer. Dynamic light scattering (DLS) and ζ potential measurements were performed on a Malvern Zetasizer Nano ZS instrument. ^{1}H NMR spectra were recorded on a Bruker NMR 400 DRX spectrometer at 400 MHz and referenced to the proton resonance resulting from incomplete deuteration of $CDCl_3$ (δ = 7.26) or DMSO-d_6 (δ = 2.50). Flow cytometry data was collected on an LSR-Fortessa 4-15 (BD Biosciences, USA) and analyzed by FlowJo software (Tree Star, USA). Confocal laser scanning microscopy (CLSM) images were collected on a Leica Stellaris 8 laser scanning confocal microscope at the University of Chicago Integrated Light Microscopy Facility and analyzed with ImageJ software (NIH, USA). The histological slides were scanned on a CRi Pannoramic SCAN 40x whole slide scanner by Integrated Light Microscopy Core in the University of Chicago and analyzed with the QuPath-0.2.3 software.[1] The absorbance and fluorescence signals from well plates were read by a BioTek Synergy HTX microplate reader. The RNA concentration was measured with a NanoDrop™ Eight Spectrophotometer (ThermoFisher).

PBS was purchased from ThermoFisher. Trypsin-EDTA solution was purchased from the American Type Culture Collection (ATCC, Rockville, MD). 3-(4,5-Dimethylthiazol-2-yl)-5-(3-carboxymethoxyphenyl)-2-(4-sulfo-phenyl)2H-tetrazolium (MTS) was purchased from Promega (USA). Murine colorectal carcinoma CT26 cells and murine triple negative breast cancer cell line

4T1 were purchased from ATCC. CT26 and 4T1 cells were cultured in RPMI-1640 (Corning, USA) (Gibco, USA). RPMI-1640 media was supported with 10% fetal bovine serum (VWR, USA), 100 U/ml penicillin G sodium and 100 μg/ml streptomycin sulphate. The cells were kept in a humidified atmosphere containing 5% CO_2 at 37°C. BALB/c breeders were obtained from Charles River Laboratories (USA) and bred in house at the animal facility at the University of Chicago. BALB/c mice with an age of 6-8 weeks were used for in vivo experiments. The study protocol was reviewed and approved by the Institutional Animal Care and Use Committee (IACUC) at the University of Chicago (PHS Assurance #D16-00322 (A3523-01)). The Human Tissue Resource Center at the University of Chicago provided the histology related services for this study. SiRNA duplexes were provided by Santa Cruz Biotechnology, Inc. and Integrated DNA Technologies.

Control siRNA-A (sc37007) (20-25 bp) consists of a scrambled sequence that does not lead to the specific degradation of any cellular message, serving as a universal negative control.

Survivin siRNA (SUR) is a pool of 3 different siRNA duplexes, with all sequences provided in 5′ → 3′ orientation.

SUR A:

• Sense: CCUUCCUCACUGUCAAGAATT

• Antisense: UUCUUGACAGUGAGGAAGGTT

SUR B:

• Sense: GAGACCAACAACAAGCAAATT

• Antisense: UUUGCUUGUUGUUGGUCUCTT

SUR C:

• Sense: CUACCCGUCAGUCAAUUGATT

• Antisense: UCAAUUGACUGACGGGUAGTT

TGF-β siRNA (TGF)

• Sense: GAAGCGGACUACUAUGCUAUU

• Antisense: UUCUUCGCCUGAUGAUACGAU-P

HIF-1α siRNA (HIF)

• Sense: CUGAUAACGUGAACAAAUATT

• Antisense: UAUUUGUUCACGUUAUCAGTT-3

TRI is a pool of SUR, TGF and HIF siRNA duplexes.

S1.2. Synthesis and Characterization of Molecules and Materials

Synthesis of 4-carboxy-*N,N,N*-trimethylbenzenaminium chloride (TMBA)

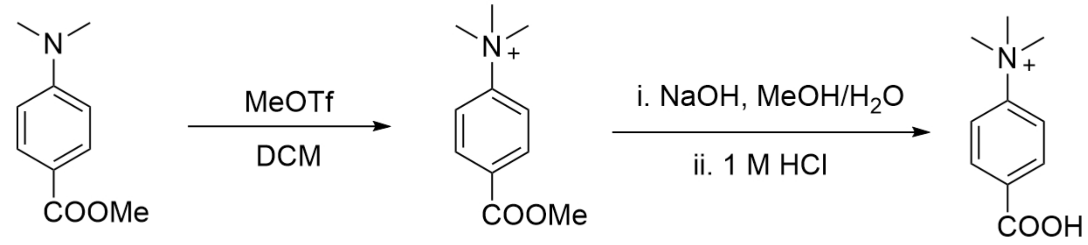

1 2 TMBA

240 mg of methyl 4-(dimethylamino)benzoate (**1**) was first dissolved in dichloromethane (DCM, 5 mL) and mixed with 286 mg of methyl triflate. The solution was stirred at room temperature for 24 hours to afford white precipitates, which were collected to give pure 4-(methoxycarbonyl)-*N*,*N*,*N*-trimethylbenzenaminium salt (**2**) as the product (456 mg, 87% yield).

343 mg of **2** was dispersed in methanol (12 mL), and 1M NaOH aqueous solution (4 mL) was added to the mixture dropwise. After stirring for 24 hours, 1M HCl was added to the solution to acidify the mixture to pH = 1. The solvent was then evaporated to give white crystals. Acetone was added to dissolve the desired TMBA product. The obtained filtrate was collected, and acetone was evaporated under vacuum to give the pure product TMBA in a quantitative yield.

Synthesis of Hf-DBP, Hf-MOL and CMOL

5,15-di(p-benzoato)porphyrin (H_2DBP) was synthesized according to the literature report.[2] Hf-DBP MOL was synthesized by adding 2 mg $HfCl_4$, 1 mg H_2DBP, 8.5 µL propionic acid (PA), 5 µL H_2O and 1 mL dimethylformamide (DMF) to a one-dram vial. The vial was placed at an 80 °C oven for 1 day. The resultant purple particles of Hf-DBP MOL were collected by centrifugation and washed with DMF and ethanol and stored as ethanol dispersions.

Trifluoroacetic acid (TFA) modification of Hf-DBP was conducted according to previous reports.[3] Briefly, a Hf-DBP suspension in ethanol was washed sequentially with anhydrous acetonitrile and toluene. The Hf-DBP suspension was degassed with N_2, and 5 mL of Hf-DBP in toluene (Hf concentration = 2 mM) was added and then treated with 20-fold trimethylsilyl trifluoroacetate (TFA-TMS) over PA. The mixture was stirred at room temperature for 16 hours. The resultant Hf-MOL was then centrifuged and washed with acetonitrile and ethanol sequentially. The replacement of PA by TFA was demonstrated by ^{1}H NMR spectroscopy.

To prepare cationic Hf-MOL (CMOL), 10 mL Hf-MOL was dispersed in DMF at a Hf concentration of 2 mM, and 10 mg of 4-carboxy-*N,N,N*-trimethylbenzenaminium was added to the suspension and stirred overnight. The resulting CMOL was collected by centrifugation, washed with ethanol, and stored in ethanol in a 4 °C refrigerator. The loading amount was quantified by NMR. For NMR analysis, 1 mg of the sample was dried in a vacuum overnight and dispersed in a mixture of 500 µL DMSO-D_6 and 50 µL of D_3PO_4. 50 µL of D_2O was then added

to the mixture, vortexed, and measured by NMR. For ζ potential measurement, Hf-DBP and CMOL were redispersed in deionized water at a Hf concentration of 20 μM. 1 mL of the sample was added to the DTS1060 cell and measured on a Malvern Zetasizer Nano ZS instrument.

S1.3. siRNA Loading Efficiency and Release Profiles of CMOL@siRNA

CMOL at a Hf concentration of 3, 15, and 30 μM was co-incubated with 120 nM of siRNA for 30 mins and then the mixture was centrifuged. The supernatant was kept for NanoDrop Spectrophotometer measurement to determine the concentration of remaining siRNA. For release profiles, CMOL@siRNA was freshly prepared and redispersed in pH=5.5 or pH=7.4 solutions in 1.5 mL Eppendorf (EP) tubes (3 replicates for each time point), respectively. The EP tubes were transferred to a 37 °C incubator. The supernatants were collected at 0, 1, 4, 8 and 24 h by centrifugation at 3,000 g and directly subjected to NanoDrop for quantification.

S1.4. X-ray Irradiation

For test tube and in vitro experiments, an RT250 orthovoltage X-ray irradiator (Philips, USA) with a fixed setting at 250 kVp, 15 mA, and a built-in 1 mm Cu filter was used. The X-ray dose rate of RT250 was 0.02564 Gy/second. For irradiating animals *in vivo*, an X-RAD 225 image-guided biological irradiator (Precision X-ray Inc., USA) was used with voltage at 225 kVp, current at 13 mA, 0.3 mm Cu filter, and 15 mm collimator. The X-ray dose rate of X-RAD 225 was 0.04167 Gy/second. The X-ray dosimetry was calibrated with an ionization chamber.

S1.5. In Vitro Studies

Cell viability assay

4T1 cells were seeded in 96-well plates at a density of 10,000 cells/well and cultured overnight. Hf-DBP or CMOL was added at a Hf concentration of 0, 7.5, 15, 30, 60, and 125 μM and incubated for 24 hours (n = 4) followed by MTS assay. IC_{50} values of all treatment groups were fitted with the non-linear regression curves in Graghpad software.

4T1 or CT26 cells were seeded in 96-well plates at a density of 10,000 cells/well and cultured overnight. CMOL, SUR, TRI, CMOL@SUR, or CMOL@TRI was added at a Hf concentration of 0, 15, 30 and 60 μM. For siRNA, the concentrations were 0, 60, 120, and 240 nM. The cells were incubated for 48 hours (n = 4) followed by MTS assay. IC_{50} values of all treatment groups were fitted with the non-linear regression curves in Graghpad software.

Cellular uptake

4T1 cells were seeded in 24-well plates at a density of 5×10^4 cells/well and incubated overnight. CMOL@siRNA^{FITC} was added at a Hf concentration of 15, 30, 60, 100 μM and an siRNA^{FITC} concentration of 120 nM (n = 3). The cells were incubated at 37 °C for 24 hours. Then the medium was aspirated, and the cells were washed with PBS three times, trypsinized, and collected by centrifugation at 300 g for 3 minutes and analyzed by flow cytometry. The

fluorescence intensity of FITC was measured using the FITC channel (ex. 488 nm / em. 525 nm).

To study lysosomal escape, free $siRNA^{FITC}$ or CMOL@$siRNA^{FITC}$ with 120 nM siRNA and 30 μM CMOL (based on Hf) was added to 4T1 cells and incubated for 4 hours. The cells were stained with Hoechst and lysotracker for 30 minutes. Afterwards, the cells were washed with PBS three times, exchanged with warm phenol-red-free RPMI-1640 medium, and mounted for confocal imaging immediately using a Leica Stellaris 8 microscope.

Real-time quantitative polymerase chain reaction (RT-qPCR)

Total RNA was extracted by using RNeasy Plus Mini Kit (QIAGEN, Cat# 74136). cDNA was then synthesized by using High-Capacity cDNA Reverse Transcription Kit (Thermo Fisher Scientific, Cat# 4368814). RT-qPCR using SYBR Green PCR Master Mix (Thermo Fisher Scientific, Cat# 43-687-02) was performed in QuantStudio 3 (Applied Biosystems) according to the manufacturer's instruction. The specific primers for RT-qPCR are as follows:

Survivin forward, 5′-GAGGCTGGCTTCATCCACTG-3′

Survivin reverse, 5′-ATGCTCCTCTATCGGGTTGTC -3′

HIF-1α forward, 5′-TCTCGGCGAAGCAAAGAGTC -3′

HIF-1α reverse, 5′-AGCCATCTAGGGCTTTCAGATAA -3′

GAPDH forward, 5′-AGGTCGGTGTGAACGGATTTG-3′

GAPDH reverse, 5′-TGTAGACCATGTAGTTGAGGTCA-3′

GAPDH was chosen as an endogenous control.

Gene expression was calculated by using the $2^{-\Delta\Delta Ct}$ method and is shown as fold change relative to the control.

Western blot analysis

All antibodies used in western blot experiments were purchased from Cell Signaling Technology or ThermoFisher. All buffers, assays, and XCell SureLock™ Mini-Cell were from ThermoFisher. The mini trans-blot electrophoretic transfer cell was from Bio-Rad, and the FluorChem R system was from ProteinSimple. Cells were lysed by RIPA buffer with protease and phosphatase inhibitor cocktail following the manufacturer's specifications. The proteins in the supernatant were collected by centrifugation at 14000 g, and the concentrations were measured and normalized by BCA assay. The proteins were denatured and reduced by NuPAGE™ LDS sample buffer with 50 mM DTT, and then heated to 80 °C for 10 min. 10 to 20 μg of samples were loaded on NuPAGE™ 4 to 12%, Bis-Tris gel for electrophoresis on a XCell SureLock™ Mini-Cell (200V, 35-50 minutes), and electrotransferred to PVDF membrane (300 mA, 70 min) on a mini trans-blot electrophoretic transfer cell. The membrane was blocked by TBST with 5% non-fat dry milk at room temperature for 1 hour and incubated with primary antibody solution in TBST with 5% BSA at 4°C overnight (Phospho-histone H2A.X (Ser139) (20E3) rabbit mAb #9718, 1:2000; Survivin (71G4B7) rabbit mAb #2808, 1:1000; HIF1-alpha recombinant rabbit mAb (BL-124-3F7), 1:1000; TGF beta-1 recombinant rabbit mAb (PD00-17), 1:1000. The

membrane was washed with TBST and incubated with secondary antibody with HRP conjugate in TBST with 5% BSA at room temperature for 1 hour (anti-rabbit IgG, HRP-linked antibody #7074, 1:5000; anti-mouse IgG, HRP-linked antibody #7076, 1:5000). The membrane was again washed with TBST and Pierce™ ECL western blotting substrate was added. The chemiluminescence was then recorded by a FluorChem R system.

In vitro total ROS detection by DCFDA assay

Total ROS generation in vitro was measured by 2',7'-dichlorodihydrofluorescein diacetate (DCFDA) assay kit (ThermoFisher, USA) following the vendor's protocol. Briefly, 4T1 cells were seeded in a 6-well plate and cultured overnight. Hf-DBP or CMOL at an equivalent Hf concentration of 30 μM was added to the wells and the cells were incubated for 8 hours. The cells were then washed with DPBS twice and exchanged with 10 μM DCFDA in prewarmed DMEM medium. The cells were incubated in a 37°C incubator for 45 minutes and then irradiated with 0 or 4 Gy of X-ray. The cells were then washed by DPBS and detached by cell scraper (Fisher Scientific) in FACS buffer (0.5% BSA, 2 mM EDTA, and 0.05% NaN_3 containing DPBS). The single cell suspension was analyzed by an LSR Fortessa 4-15 (BD Biosciences) flow cytometer at the FITC channel.

In vitro $^{\cdot}OH$ generation by γ-H_2AX assay

γ-H_2AX, a protein that is phosphorylated after oxidation by hydroxyl radicals to induce DNA damage repair, has been used as a sensitive biomarker for probing DNA double strand breaks. For flow cytometry, 4T1 cells were plated in 24-well plates overnight and incubated with CMOL or CMOL@TRI at a Hf concentration of 30 μM for 8 h followed by irradiation at 0 or 4 Gy X-ray. After incubation for 24 additional hours, the cells were washed with PBS, collected following fixation with 4% paraformaldehyde and permeabilization with the permeabilize buffer (PBS + 1%FBS + 0.3% Triton-X), and resuspend in diluted primary antibody Phospho-histone H2A.X (Ser139) in flow buffer (DPBS with 0.5% BSA) at 1:200 dilution and cultured for 1 h at room temperature. The cells were then washed with flow buffer twice and resuspended in 100 μl of diluted fluorophore-conjugated secondary antibody 1:2000 for 30 min at room temperature in the dark. After washing, the cells were resuspended in flow buffer and analyzed on a flow cytometer.

For CLSM analysis, 4T1 cells were plated in 35 mm tissue culture dishes overnight and incubated with CMOL or CMOL@TRI at a Hf concentration of 30 μM for 8 h followed by irradiation at 0 or 4 Gy X-ray. After incubation for 24 additional hours, the cells were washed with PBS, fixed with 4% paraformaldehyde, and blocked in blocking buffer (DPBS with 5% BSA and 0.3% FBS and 0.3% Triton-X) for 1 h. The cells were resuspended with dilute primary antibody Phospho-histone H2A.X (Ser139) in confocal buffer (DPBS with 5% FBS and 0.3% Triton-X) at 1:400 dilution overnight. Then cells were washed three times with PBS and resuspended in 100 μl of diluted fluorophore-conjugated secondary antibody 1:2000 for 1 h in the dark. After washing, the cells were analyzed on LeicaSP8 CLSM.

Colony assay

Colony assay was performed to evaluate the proliferation ability of 4T1 cells after RT treatment with TRI, CMOL, or CMOL@TRI. The cells were first seeded on 6-well plates at a density of 1 × 10^5 cells/well and further cultured overnight. TRI, CMOL, or CMOL@TRI was added to the wells at a Hf concentration of 30 μM and TRI concentration of 120 nM and incubated for 8 hours (n = 3), followed by 0, 2, 4, 6, 8, 10 Gy X-ray irradiation. Then the cells were trypsinized, counted, and reseeded in new 6-well plates at a density of 200 cells per well with 2.5 mL medium per well for a week. Once colony formation was observed, the culture medium was discarded. The plates were rinsed twice with PBS, then stained with 500 μL of 0.5% w/v crystal violet in 50% methanol/H_2O. The wells were rinsed with water three times and the colonies were counted manually.

Apoptosis assay

Apoptosis with or without RT treatment was evaluated on 4T1 and CT26 cells by flow cytometry. For apoptosis without RT irradiation, 4T1 (or CT26) cells were seeded at a density of 5 × 10^4 cells/well and cultured overnight on 24-well plates. The cells on both plates were treated with PBS, SUR, TRI, CMOL, CMOL@SUR, or CMOL@TRI at a Hf concentration of 30 μM and a total siRNA concentration of 120 nM and further incubated for 48 hours. The cells were then scraped off and stained with Alexa Fluor 488 Annexin V/dead cell apoptosis kit (ThermoFisher, USA) following the vendor's protocol for flow cytometry analysis.

For apoptosis after RT irradiation, 4T1 (or CT26) cells were seeded at a density of 1 × 10^5 cells/well and cultured overnight on 24-well plates. The cells on both plates were treated with PBS, CMOL, CMOL@SUR, or CMOL@TRI at an equivalent Hf concentration of 30 μM and a total siRNA concentration of 120 nM and further incubated for 8 hours. Then the plates were irradiated with 0 or 6 Gy X-ray irradiation followed by incubation for 24 hours. The cells were then scraped off and stained with Alexa Fluor 488 Annexin V/dead cell apoptosis kit for flow cytometry analysis.

Cell cycle analysis

For cell cycle analysis without X-ray treatment, 4T1 and CT26 cells were seeded in 24-well plates at a density of 5 × 10^4 cells/well and cultured overnight. The cells were then treated with PBS, SUR, TRI, CMOL, CMOL@SUR, or CMOL@TRI at a Hf concentration of 30 μM and a total siRNA concenrration of 120 nM. After incubation for another 24 h, the cells were collected by adding 200 μL of 0.05% trypsin-containing EDTA to each well and centrifugation (300 g) for 5 min. The cells were fixed using 3 mL ice-cooled 70% ethanol overnight and then centrifuged at 300 g for 5 min. The precipitate was treated with 450 μL of Hoechst staining solution. After overnight incubation, flow cytometry analysis was performed. All experiments were performed in triplicate.

For cell cycle analysis after X-ray treatment, 4T1 and CT26 cells were seeded in 24-well plates at a density of 5 × 10^4 cells/well and cultured overnight. The cells were then treated with PBS, CMOL, or CMOL@TRI at a Hf concentration of 30 μM and a total siRNA concentration of 120 nM. After incubation for another 8 h, the cells were irradiated by 6 Gy of X-rays. After incubation for another 24 hours, the cells were treated as above for flow cytometry analysis.

S1.6 In Vivo Study

Intratumoral retention of CMOL@siRNA

A CT26 model was established in BALB/c mice by inoculating 2 × 10^6 cells/mouse subcutaneously. On day 7, $siRNA^{Cy3}$ or CMOL@$siRNA^{Cy3}$ was injected intratumorally with an equivalent $siRNA^{Cy3}$ dose of 2 nmol (n = 3). At each time point, the tumors were excised and immersed in 15 mL ep tubes filled with 2 mL 5% H_3PO_4 (on ice). The tumor tissues were then homogenized with a probe sonicator (500 W, 20 kHz) with 30% power for 1 minute on ice. The mixture was centrifuged at 2,000 g at 4 ^{o}C for 5 minutes to remove all the solid residues, and 100 µL of supernatants were collected for analysis by In Vivo Imaging System.

Anti-cancer Efficacy

For the evaluation of antitumor efficacy of CMOL@siRNA treatments, syngeneic models were established by inoculating 2×10^6 4T1 cells (or CT26 cells) onto the right flank subcutaneous tissues of C57Bl/6 mice on day 0 as the 4T1 model (or the CT26 model). When the tumors reached ~100 mm^3 in volume, PBS, CMOL, CMOL@SUR, CMOL@TRI, or PC@TRI at a Hf dose of 0.5 µmol and/or siRNA dose of 2 nmol was injected intratumorally. 24 h after injection, the mice were anaesthetized with 2% (v/v) isoflurane and the tumors were irradiated with 3 Gy X-ray per fraction (225 kVp, 13 mA, 0.3 mm-Cu filter) for a total of 6 daily fractions (4 daily fractions for the CT26 model). The tumor sizes were measured with a caliper every day where tumor volume equals (width^2 × length)/2. Body weights of the nice were also monitored every day. The mice were sacrificed when the PBS group reach the endpoint. Tumors and major organs were sectioned for hematoxylin-eosin staining (H&E), immunofluorescent TUNEL analysis, as well as immunohistochemistry staining for survivin, phospho-histone H_2A.X, TGF-β, and HIF-1α. The tumor growth inhibition index (TGI) was defined as the equation below:

$$TGI = 1 - \frac{\frac{Te}{Ts}/\frac{Ce}{Cs}}{1-\frac{Cs}{Ce}} \times 100\%$$

where Te, Ts, Ce, and Cs represent average tumor volumes of treated mice at endpoint, treated mice at starting-point, control mice at endpoint and control mice at starting-point, respectively.

S1.7 Statistical analysis

All statistical analysis was performed on Origin Lab software by pair-sample t-test or One-way Repeated Measures ANOVA method. The p values were defined as * $p<0.05$, ** $p<0.01$, *** $p<0.001$, **** $p<0.0001$ in all figures.

S1.8 Ethical statement

This research complies with all relevant ethical regulations. All work performed on animals was in accordance with and approved by the Institutional Animal Care and Use Committee at the

University of Chicago. The approved number is 72408. Animals were housed in 12 light/12 dark cycle, 65-75°F (~18-23°C), and 40-60% humidity condition. Animals were euthanized when the tumor reached 20 mm in any dimension or when they became moribund with severe weight loss or unhealing ulceration. This limit was not exceeded at any point.

S2 Supporting Figures and Tables

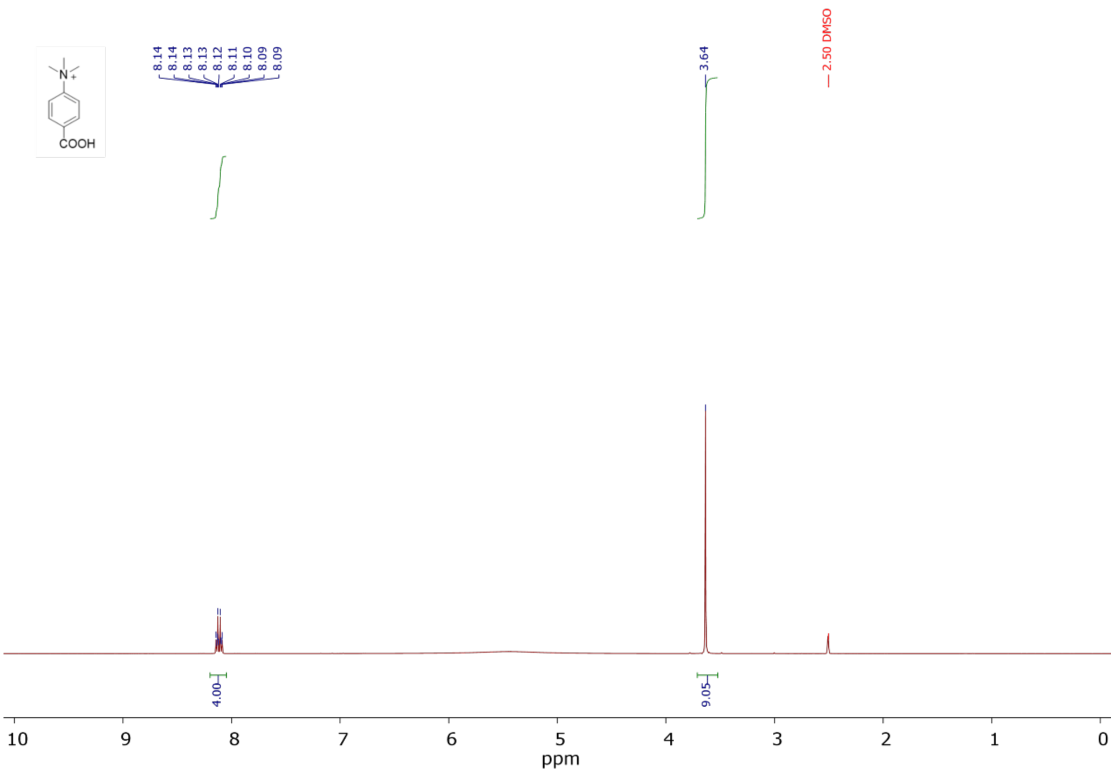

Figure S1. ^{1}H NMR of 4-carboxy-N,N,N-trimethylbenzenaminium chloride.

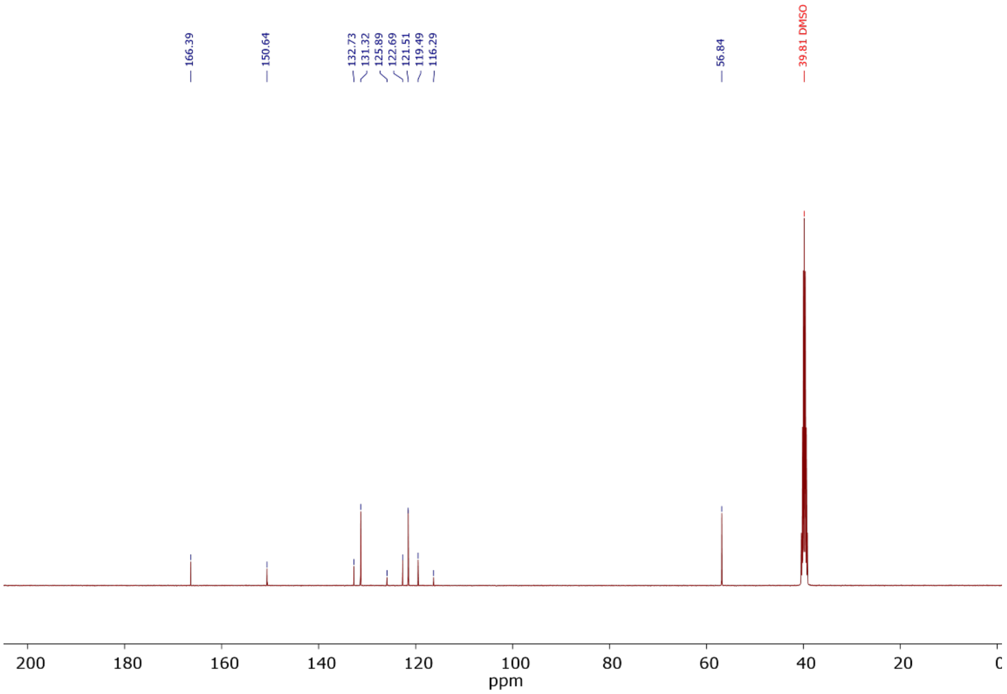

Figure S2. $^{13}C\{^1H\}$ NMR spectrum of 4-carboxy-N,N,N-trimethylbenzenaminium chloride.

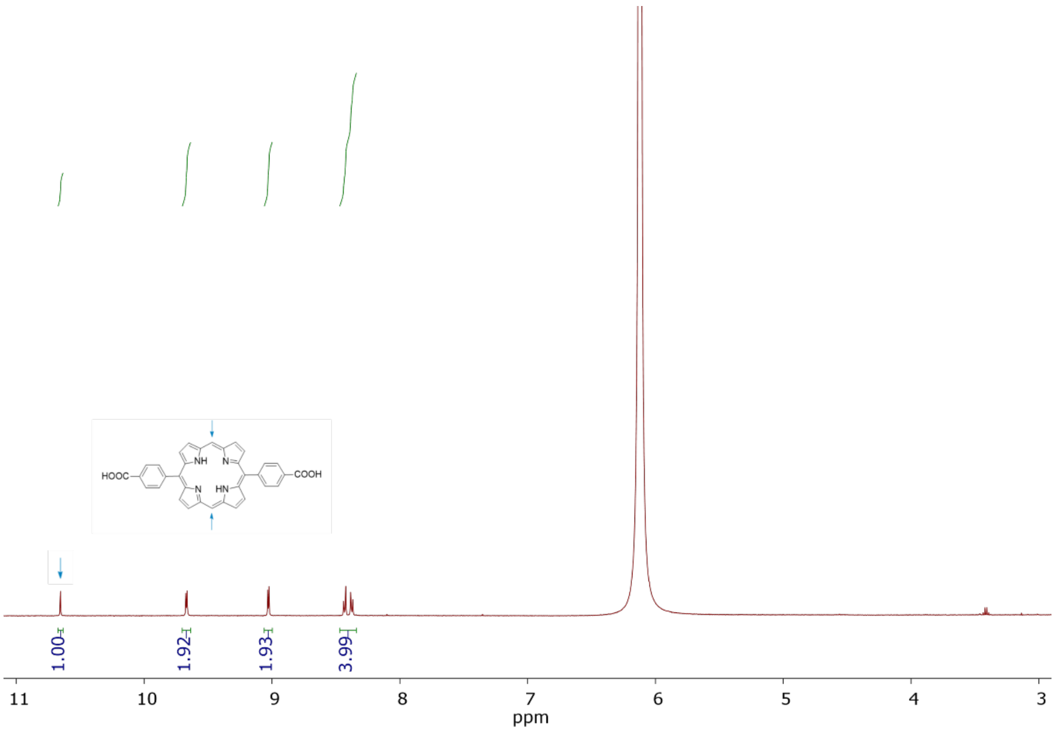

Figure S3. ^1H NMR of digested Hf-MOL.

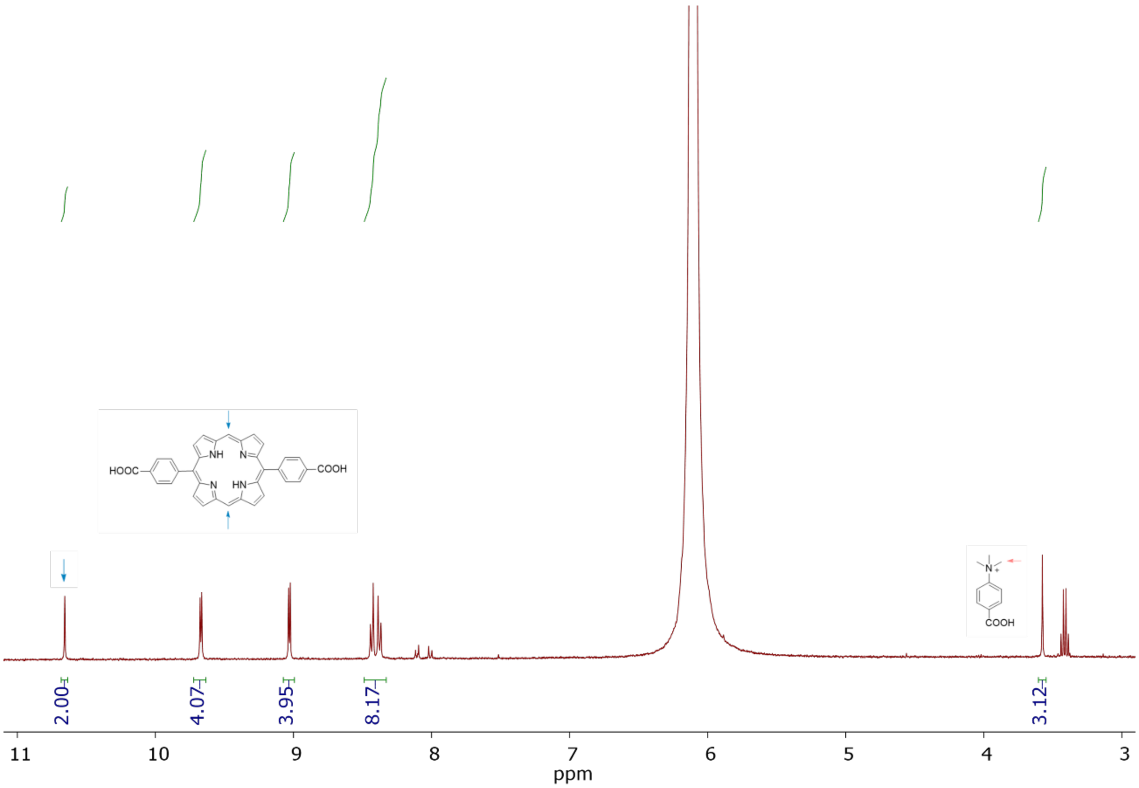

Figure S4. ^{1}H NMR of digested CMOL.

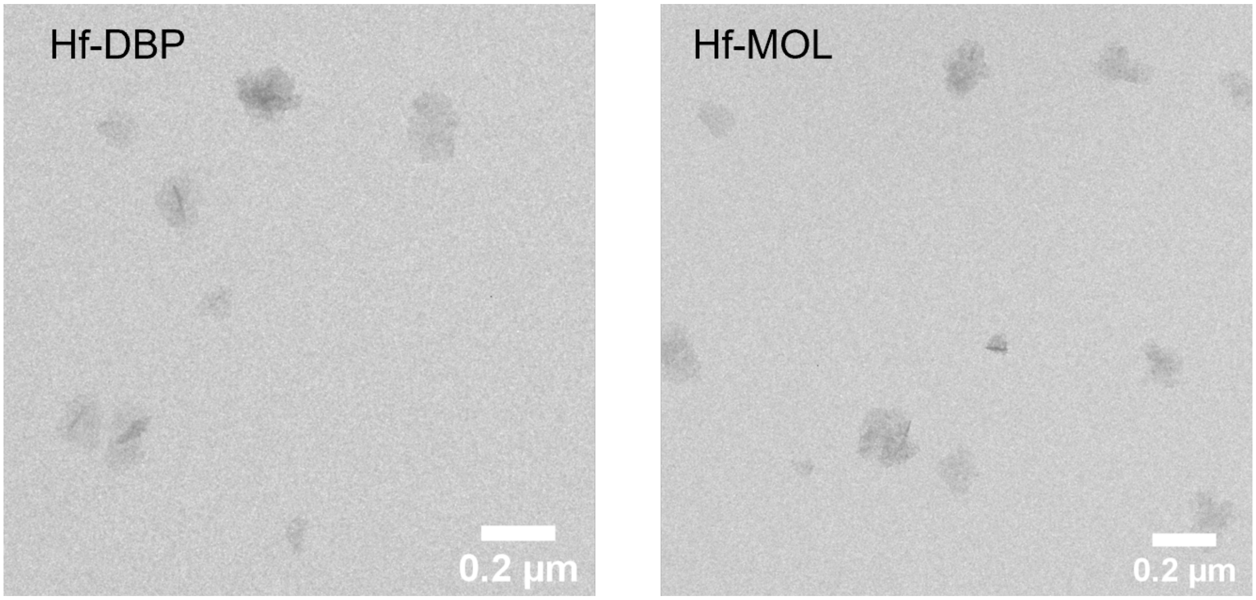

Figure S5. TEM images of PA-capped Hf-DBP (left) and TFA-capped Hf-MOL (right).

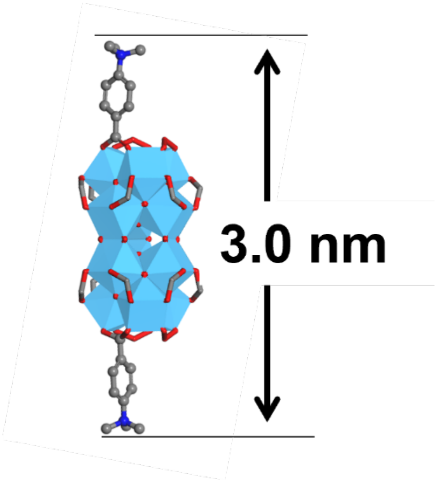

Figure S6. A view of simulated structure of TMBA-capped Hf_{12} cluster with a height of ~3.0 nm.

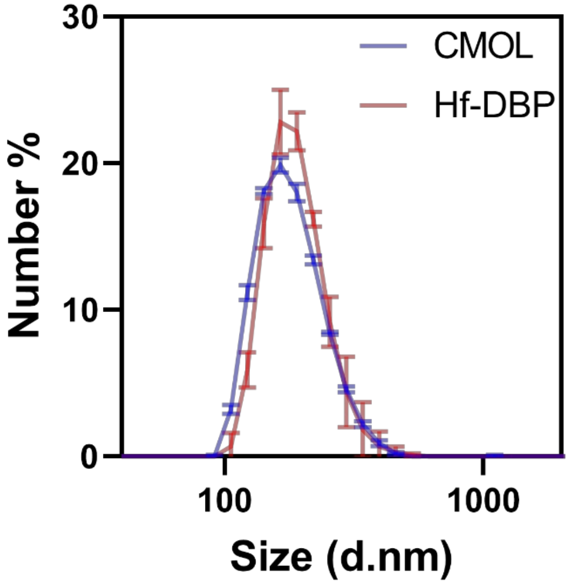

Figure S7. Dynamic scattering sizes of Hf-DBP and CMOL in water.

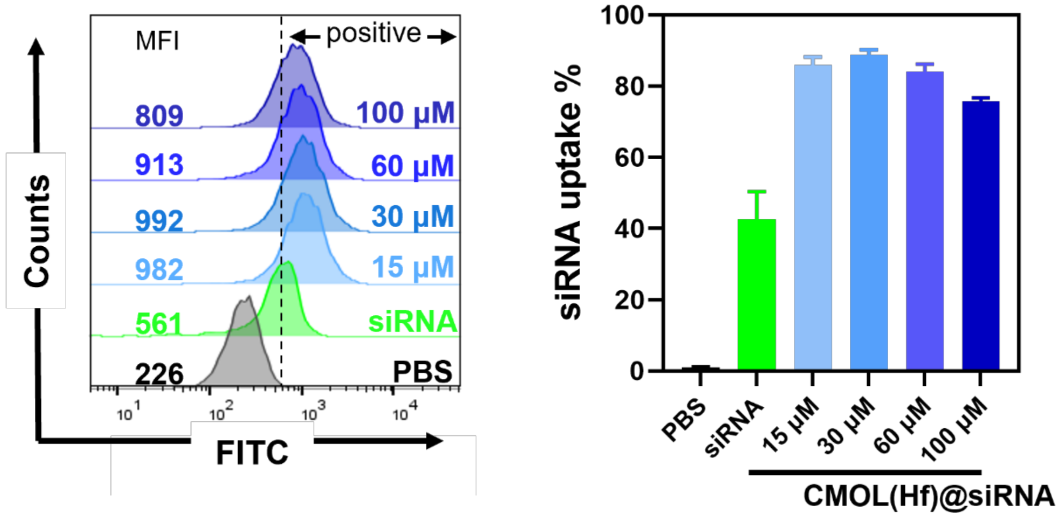

Figure S8. Flow cytometry histograms of FITC channel (left) and quantitative analysis of FITC-positive cells (right, represents the siRNA^{FITC} uptake level) after treatment with CMOL@siRNA^{FITC} at different concentrations of CMOL in 4T1 cells, n=3. The dashline represents the FITC-positive gate. The concentration of siRNA^{FITC} was 120 nM. The concentration of CMOL was 15, 30, 60, or 100 μM.

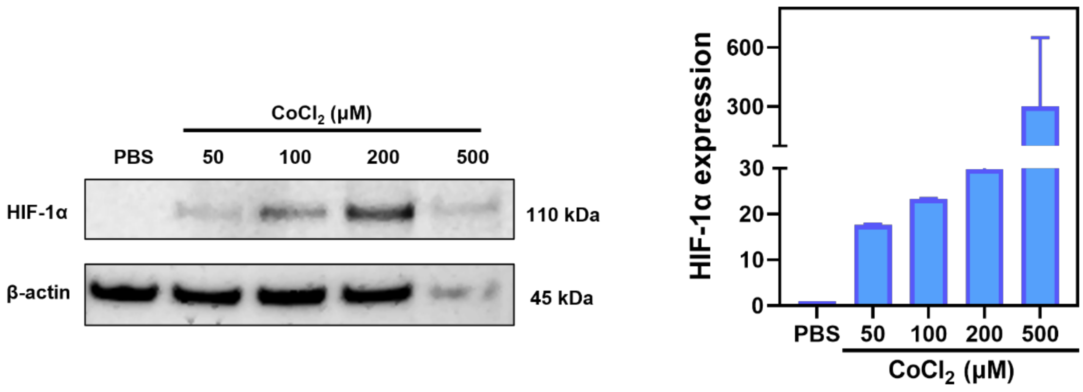

Figure S9. Western blots of HIF-1α expressions in 4T1 cells after treatment with 50, 100, 200, or 500 μM of $CoCl_2$ for 24 h.

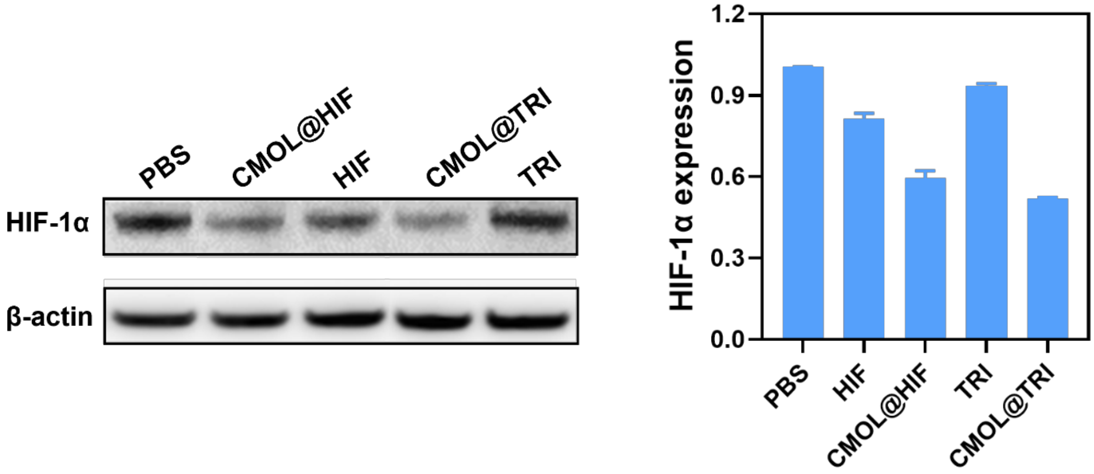

Figure S10. Western blots of HIF-1α expressions in CT26 cells after treatment with $CoCl_2$ with PBS, HIF, TRI, CMOL@HIF, CMOL@TRI, PC@HIF, or PC@TRI for 24 h.

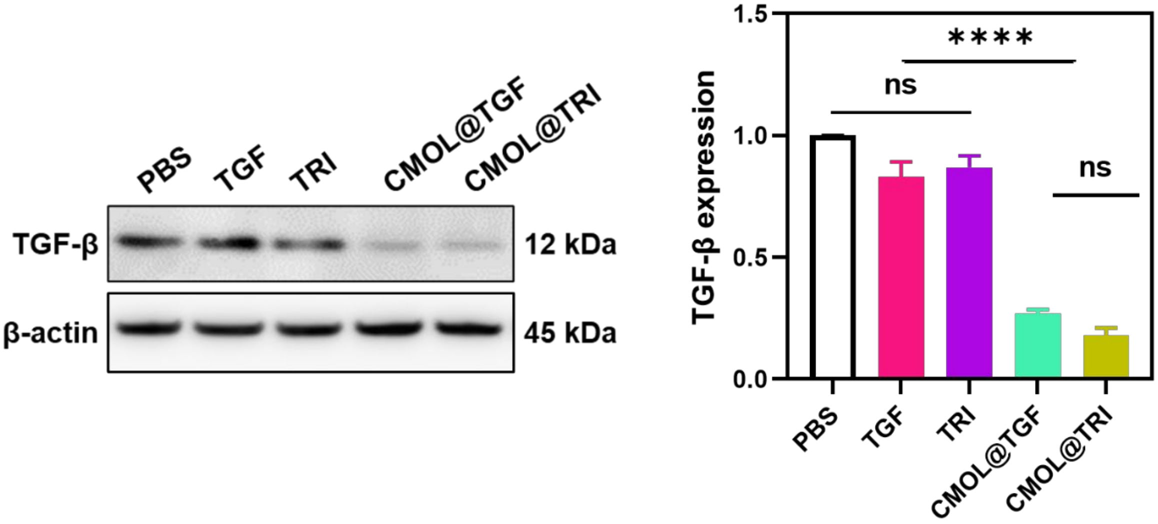

Figure S11. Western blots of TGF-β expressions in 4T1 cells after treatment with PBS, CMOL, TGF, TRI, CMOL@TGF, or CMOL@TRI for 24 h.

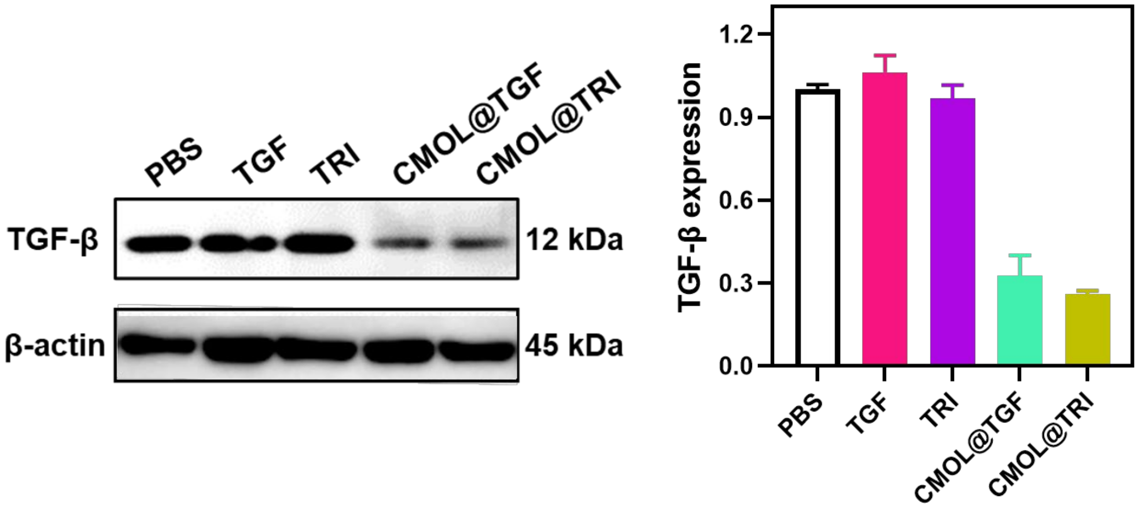

Figure S12. Western blots of TGF-β expressions on CT26 cells after treatment with PBS, CMOL, TGF, TRI, CMOL@TGF, or CMOL@TRI for 24 h.

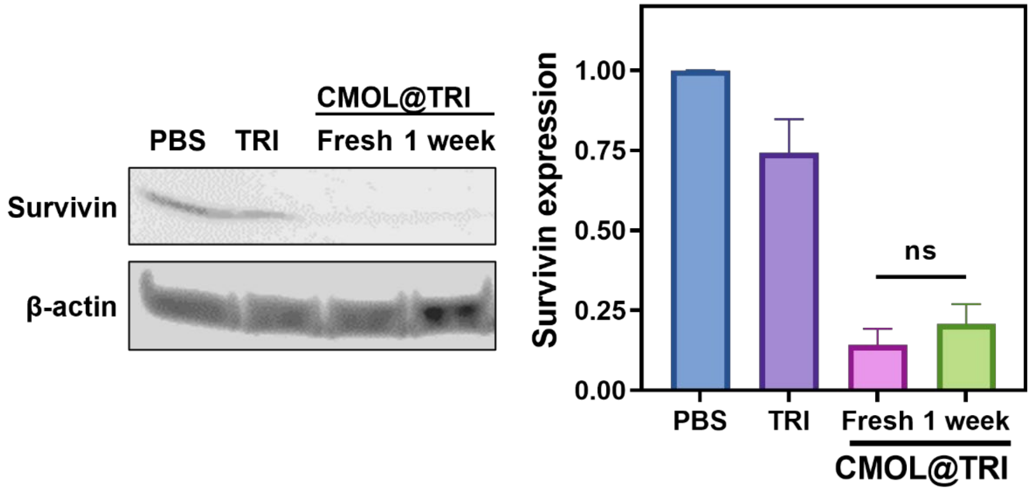

Figure S13. Western blots of survivin expressions in 4T1 cells after 24 h treatment with PBS, TRI, freshly prepared CMOL@TRI, or CMOL@TRI after being stored at 4°C for 1 week. *p<0.05, **p<0.01, ***p<0.001, ****p<0.0001.

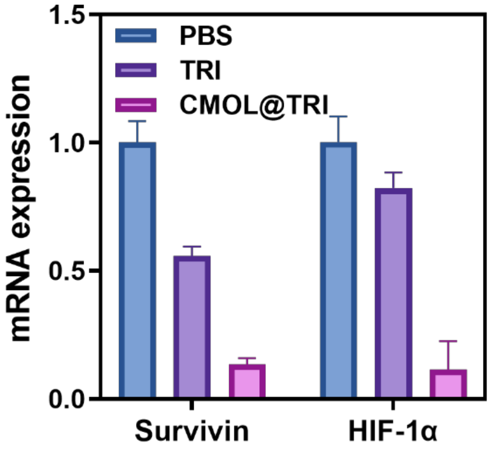

Figure S14. Relative Survivin and HIF-1α mRNA levels in 4T1 cells after treatment with PBS, TRI, or CMOL@TRI for 24 h.

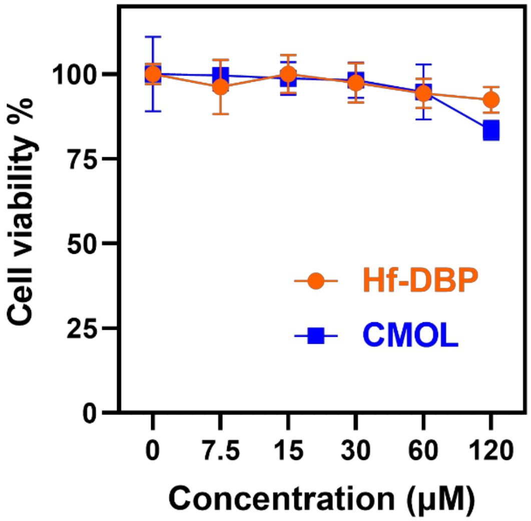

Figure S15. Viability curves (mean ± SD %) of 4T1 cells after treatment with Hf-DBP or CMOL.

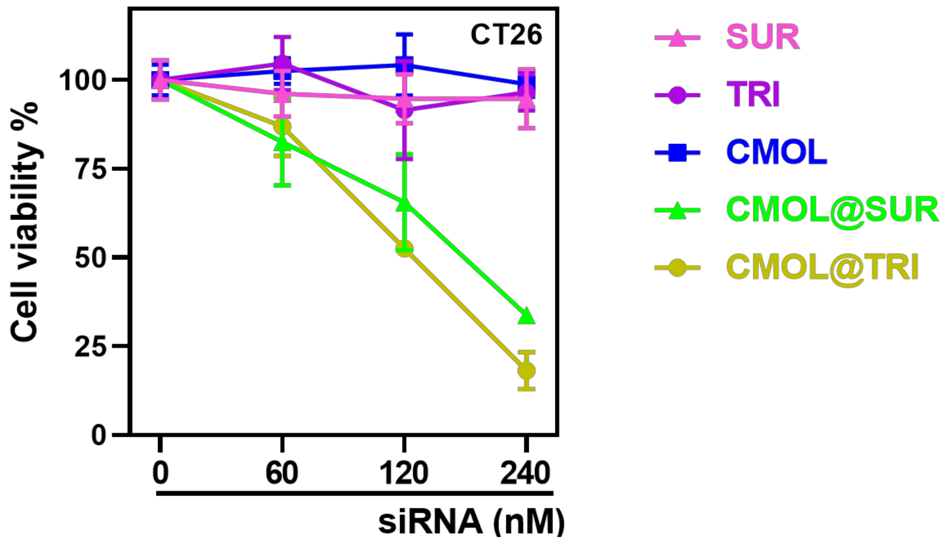

Figure S16. Viability curves (mean ± SD %) of CT26 cells after treatment with CMOL, SUR, TRI, CMOL@SUR, or CMOL@TRI.

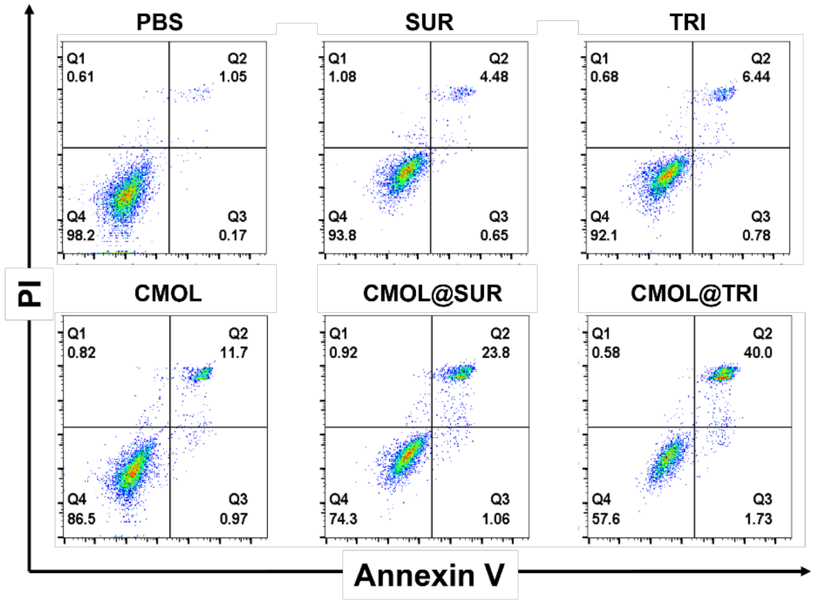

Figure S17. Apoptosis assay of 4T1 cells after treatment with CMOL, SUR, TRI, CMOL@SUR, CMOL@TRI.

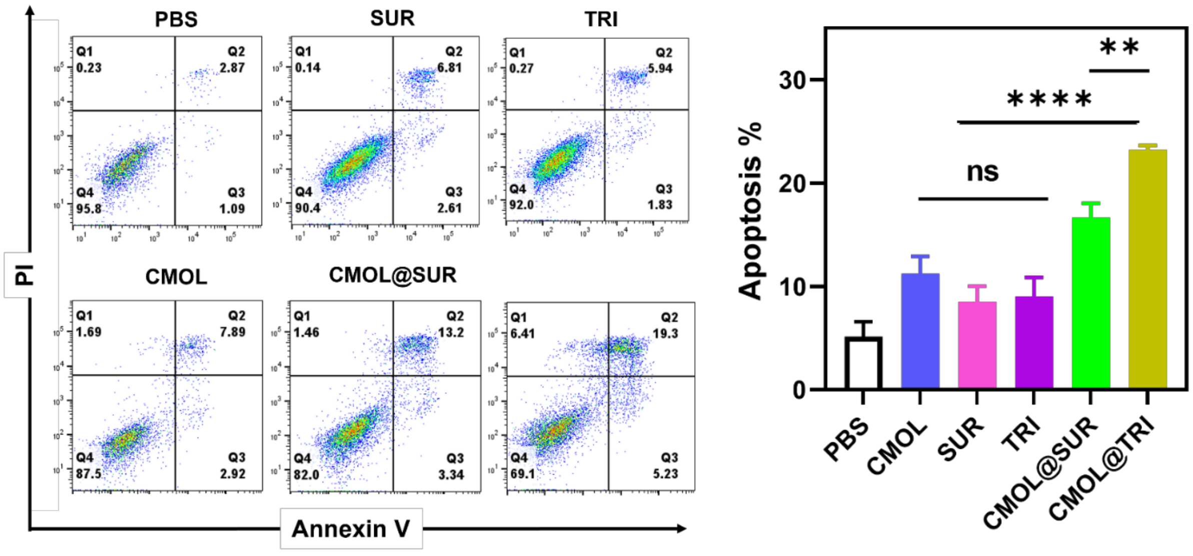

Figure S18. Apoptosis assay (left) and analysis (right) of CT26 cells after treatment with CMOL, SUR, TRI, CMOL@SUR, CMOL@TRI.

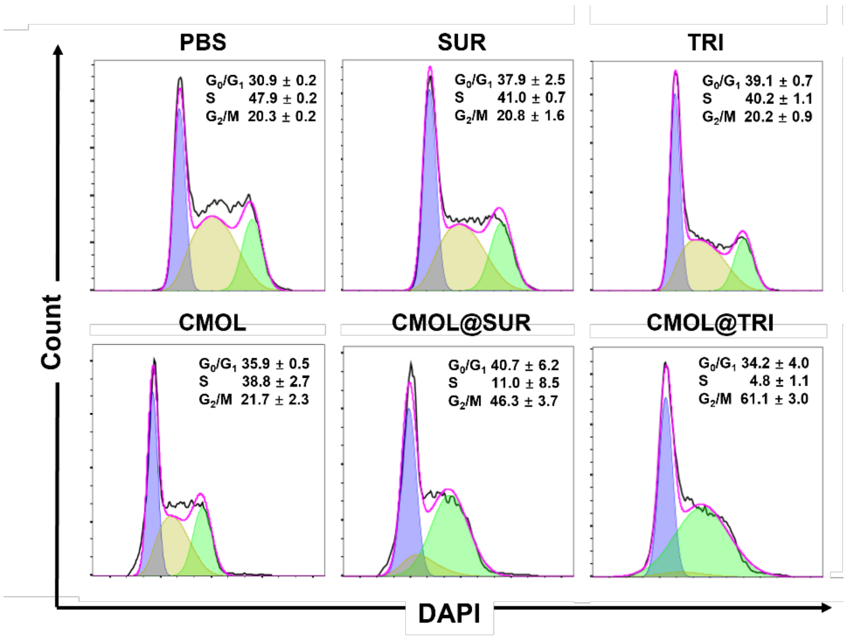

Figure S19. Cell cycle analysis of 4T1 cells after treatment with CMOL, SUR, TRI, CMOL@SUR, or CMOL@TRI.

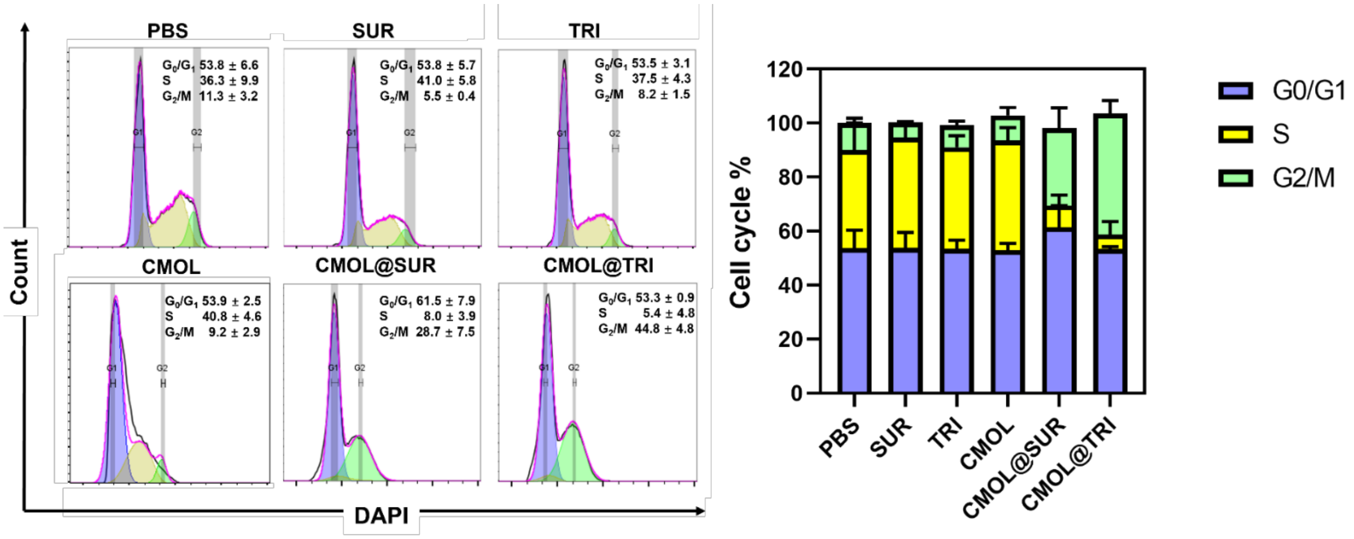

Figure S20. Cell cycle assay (left) and analysis (right) of CT26 cells after treatment with CMOL, SUR, TRI, CMOL@SUR, or CMOL@TRI.

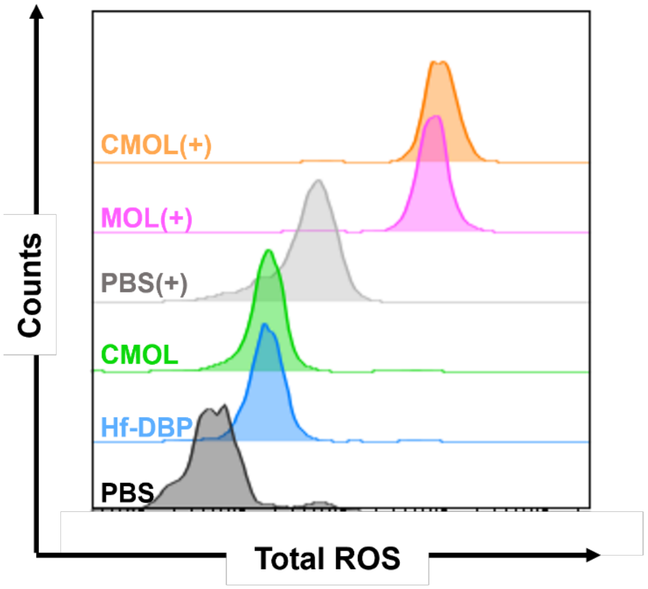

Figure S21. Flow cytometry histograms of total ROS signals in 4T1 cells after 4 Gy X-ray irradiation (n=3).

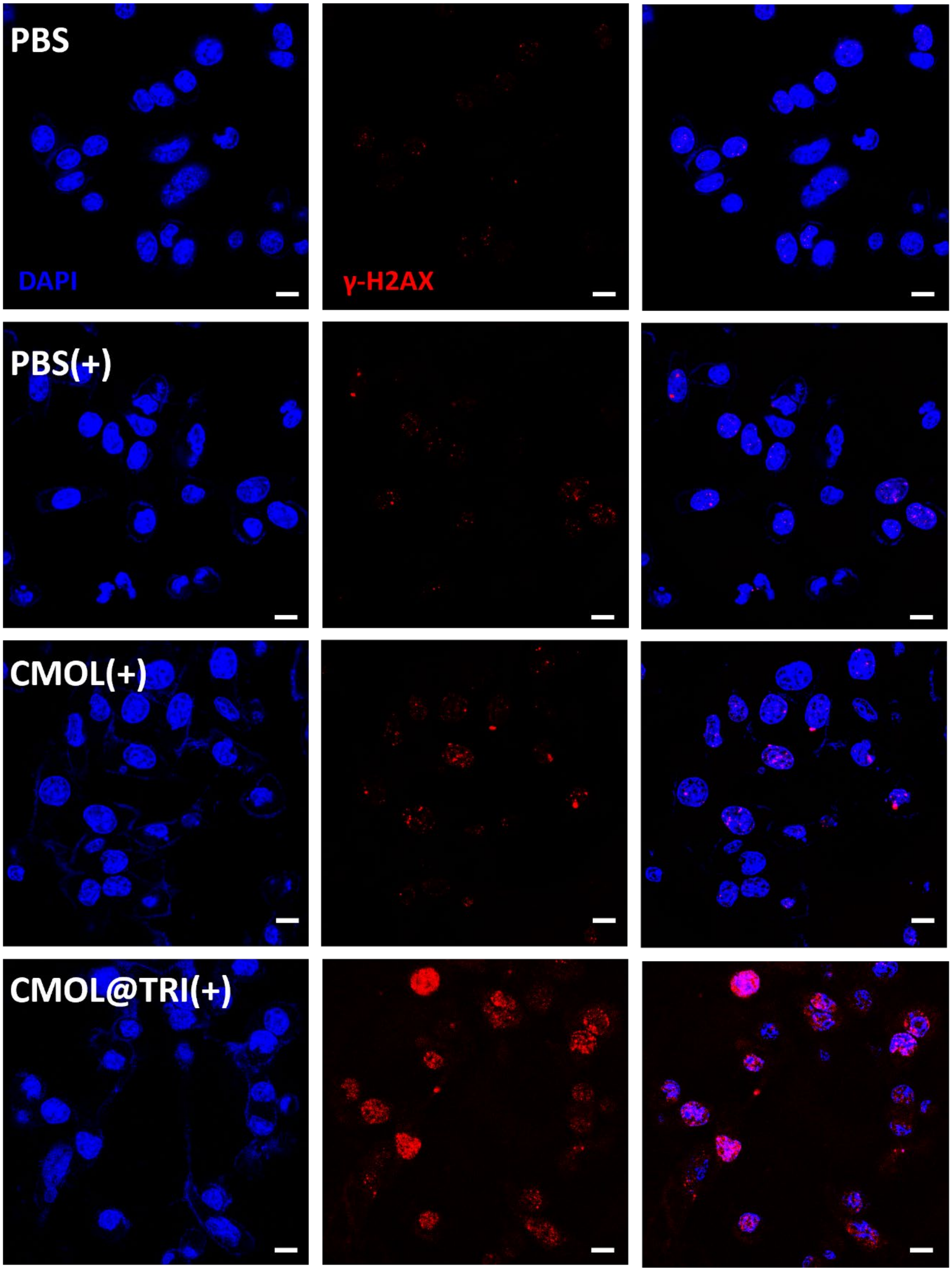

Figure S22. CLSM images of γ-H2AX in 4T1 cells after different treatments (Hoechst, blue; γ-H2AX, red; scale bar = 10 μm).

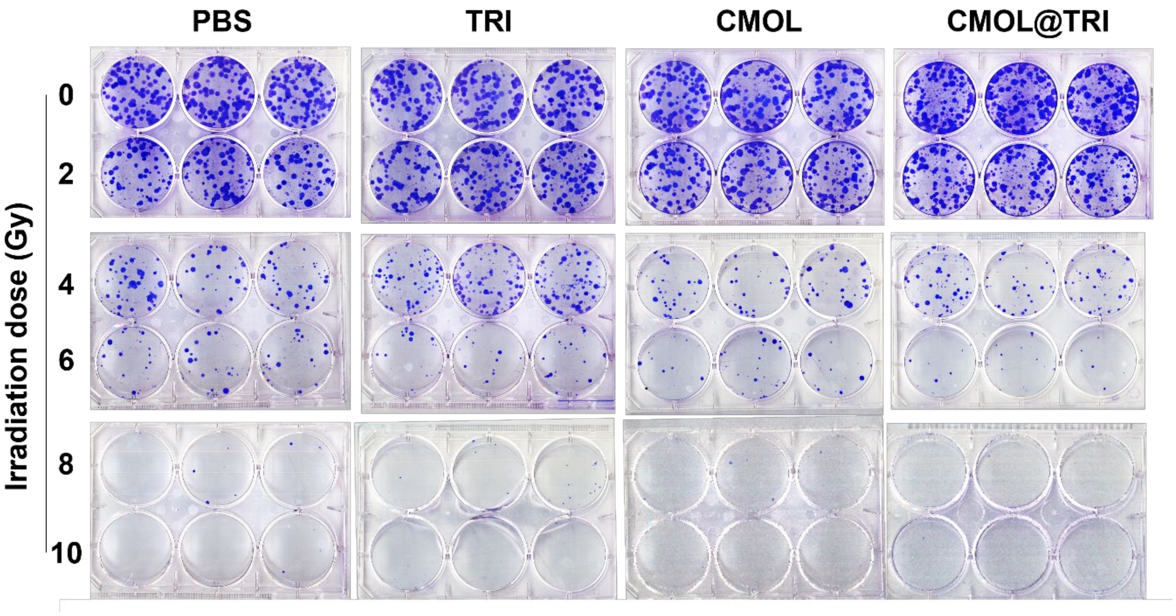

Figure S23. Representative images of 4T1 cell colonies after different treatments (n=3).

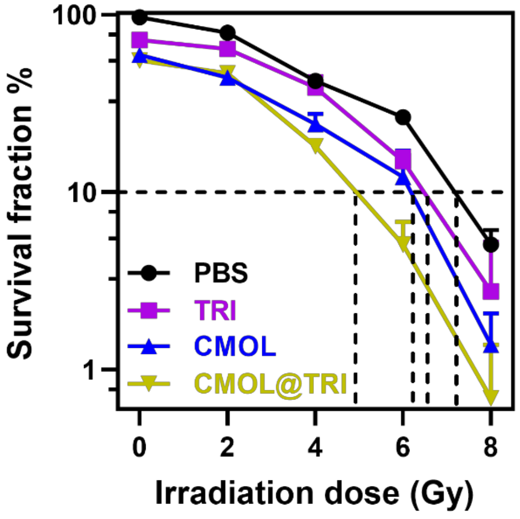

Figure S24. Survival fraction analysis of 4T1 cells after different treatments (n=3).

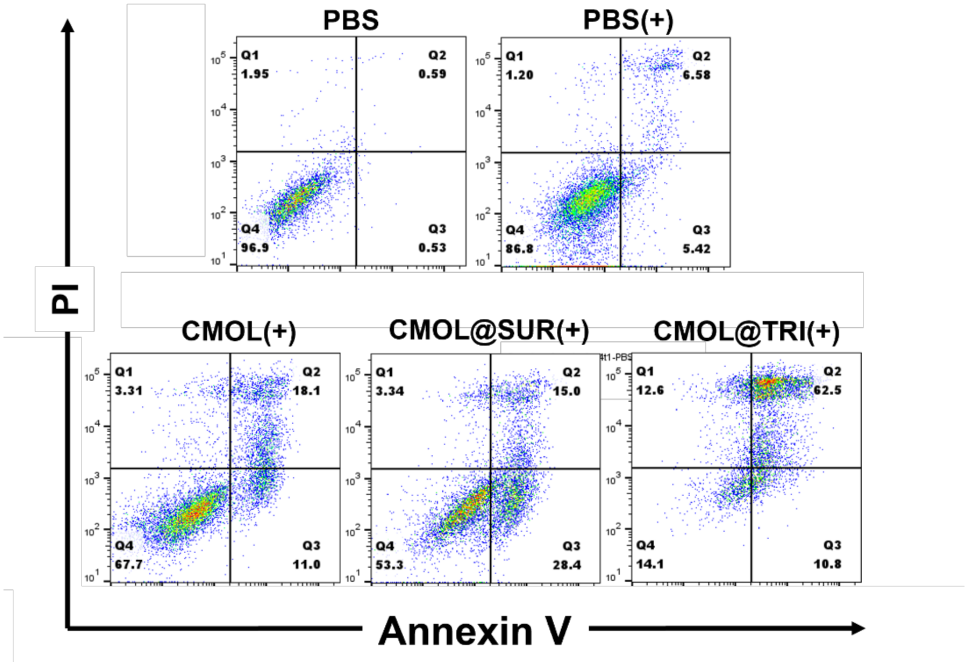

Figure S25. Apoptosis assay of 4T1 cells after treatment with PBS, CMOL, CMOL@SUR, or CMOL@TRI plus 6 Gy X-ray irradiation (n=3).

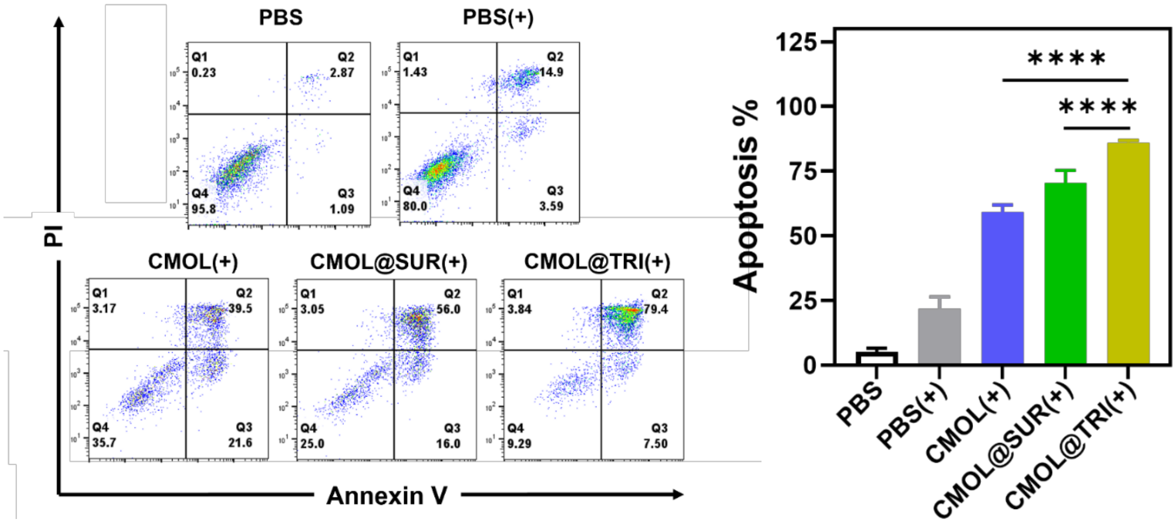

Figure S26. Cell apoptosis assay (top) and apoptotic rates (bottom) of CT26 cells after treatment with PBS, CMOL, CMOL@SUR, or CMOL@TRI plus 6 Gy X-ray irradiation (n=3).

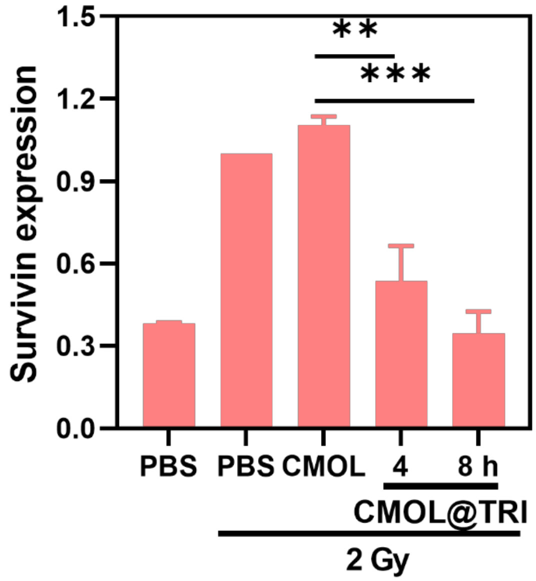

Figure S27. Western blot analysis of survivin expression in 4T1 cells upon 2 Gy of X-ray irradiation.

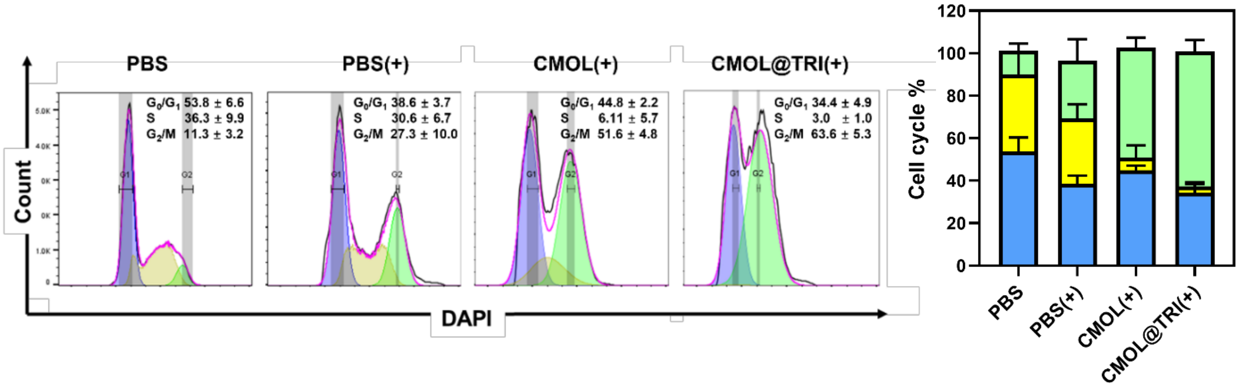

Figure S28. Cell cycle assay of CT26 cells after treatment with PBS, CMOL, or CMOL@TRI plus 6 Gy X-ray irradiation (n=3).

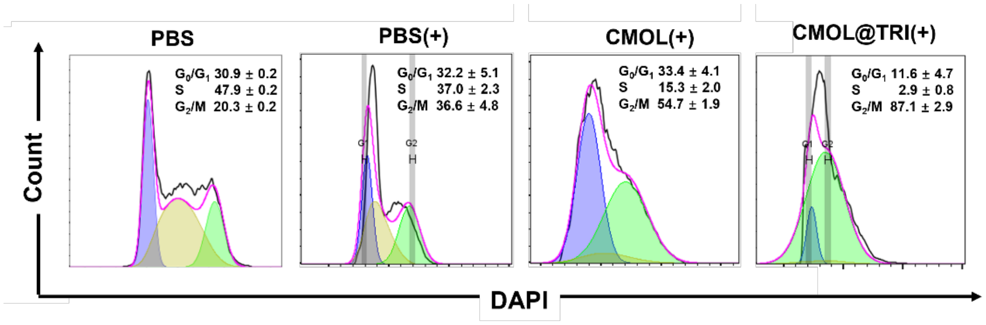

Figure S29. Cell cycle assay of 4T1 cells after treatment with PBS, CMOL, or CMOL@TRI plus 6 Gy X-ray irradiation (n=3).

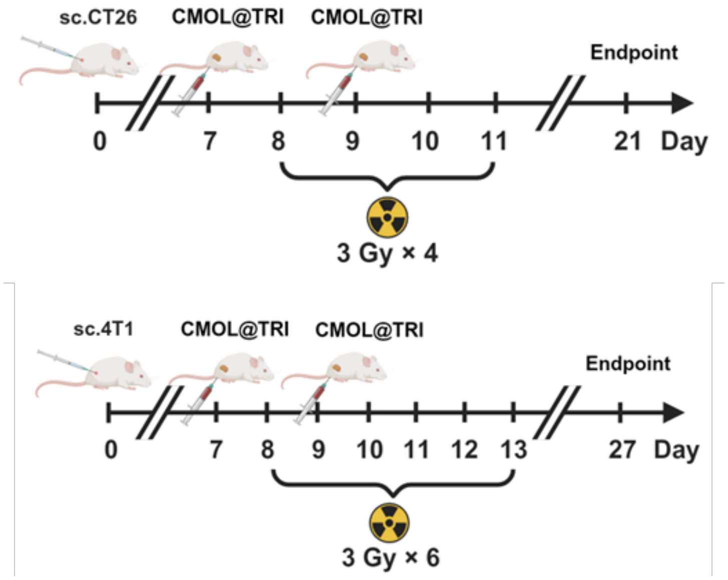

Figure S30. Tumor inoculation and treatment schedules. All controls or particles were intratumorally injected and 3 Gy X-ray per fraction was given in 4 consecutive days for CT26 tumor and in 6 consecutive days for 4T1 tumor starting on day 7 post tumor inoculation. Created with BioRender.

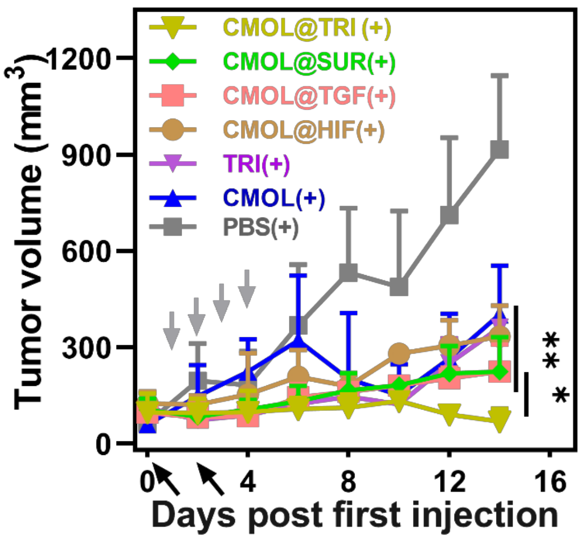

Figure S31.Tumor growth curves of subcutaneous CT26 tumor-bearing BALB/c mice after different treatments (grey arrows: 3 Gy irradiation; black arrows: intratumoral injection) (n=5).

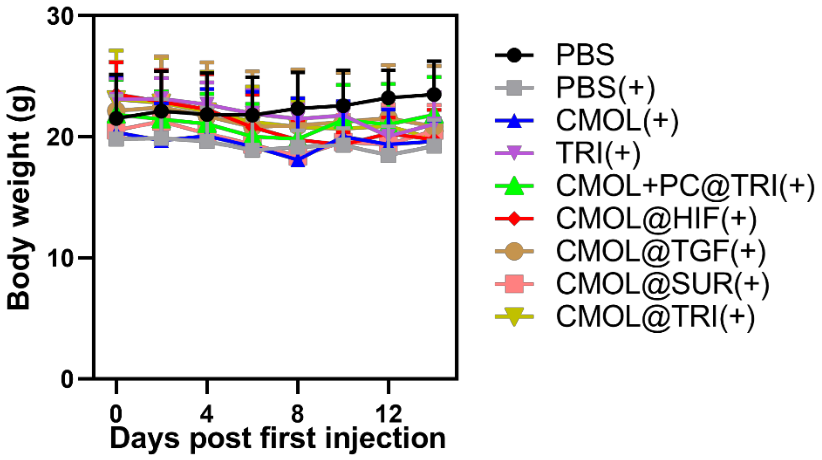

Figure S32. Body weights of subcutaneous CT26 -tumor-bearing BALB/c mice after different treatments (n=5).

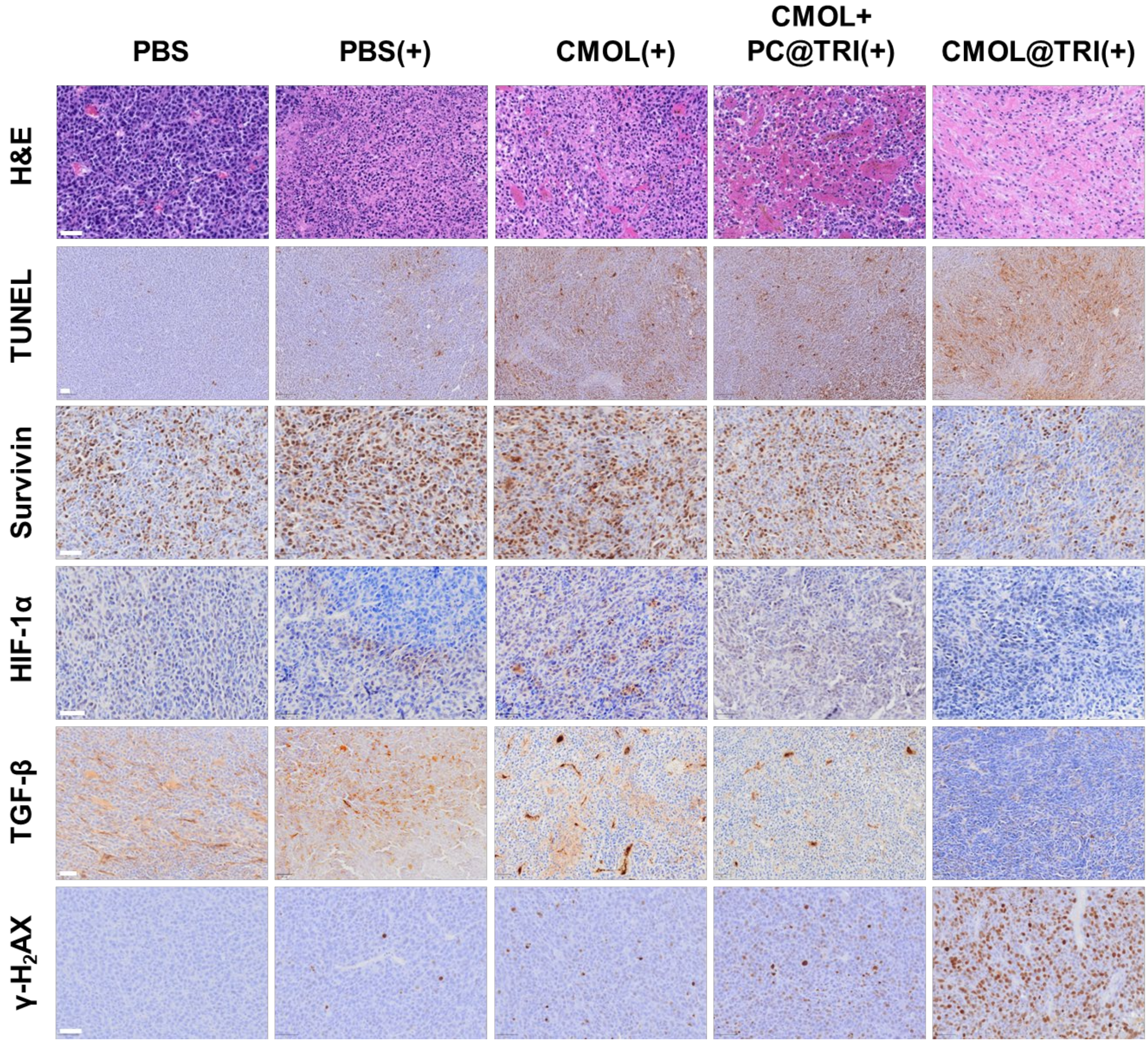

Figure S33. H&E, TUNEL, and immunohistochemistry staining of HIF-1α, TGF-β, survivin and γ-H2AX of excised CT26 tumor sections in different treatment groups (Nuclei stained by hematoxylin in blue; TUNEL positive by 3,3'-Diaminobenzidine (DAB) in red; scale bar = 50 μm).

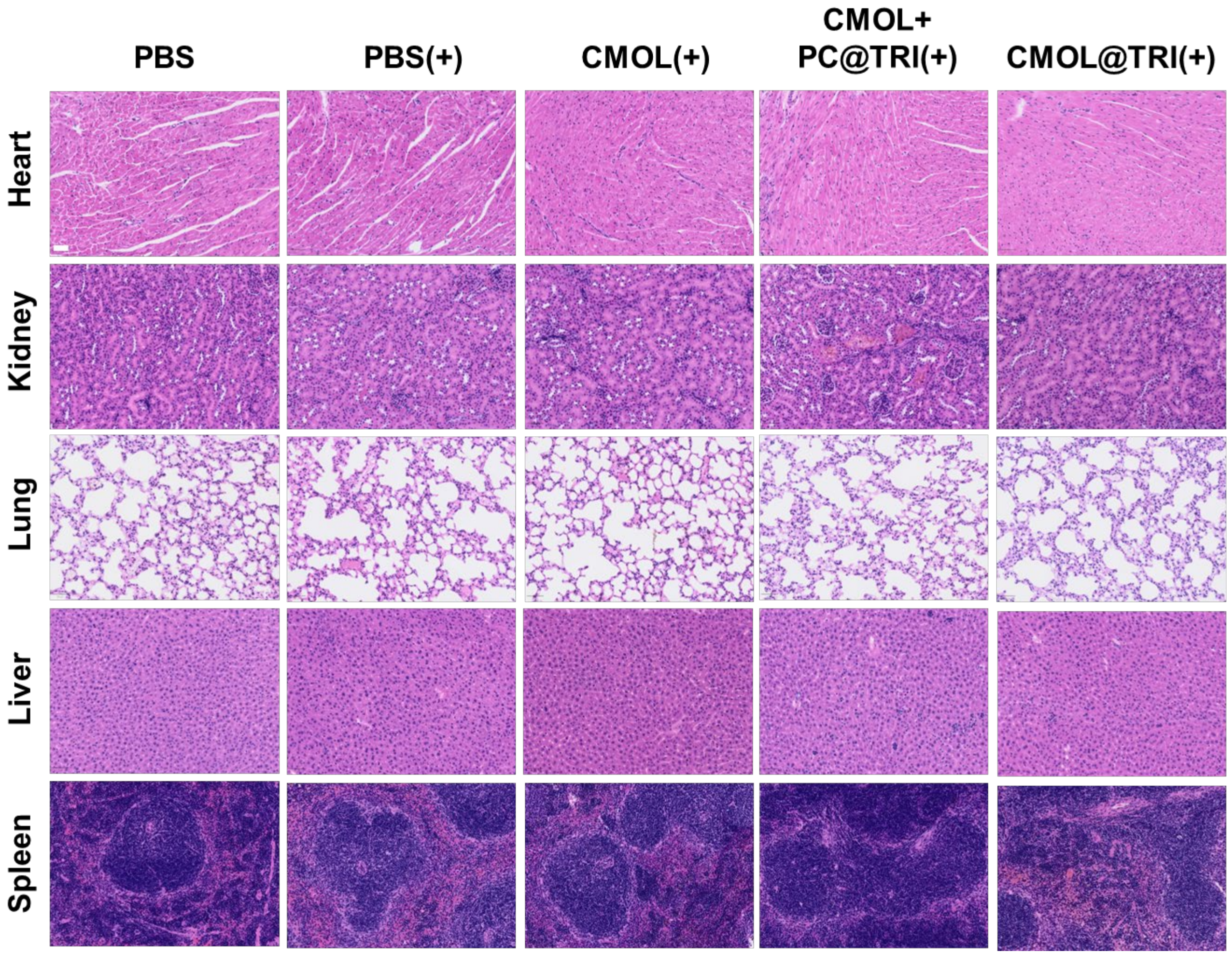

Figure S34. H&E staining of heart, lung, liver, spleen, kidney from CT26 tumor-bearing BALB/c mice in different treatment groups (Scale bar = 50 μm).

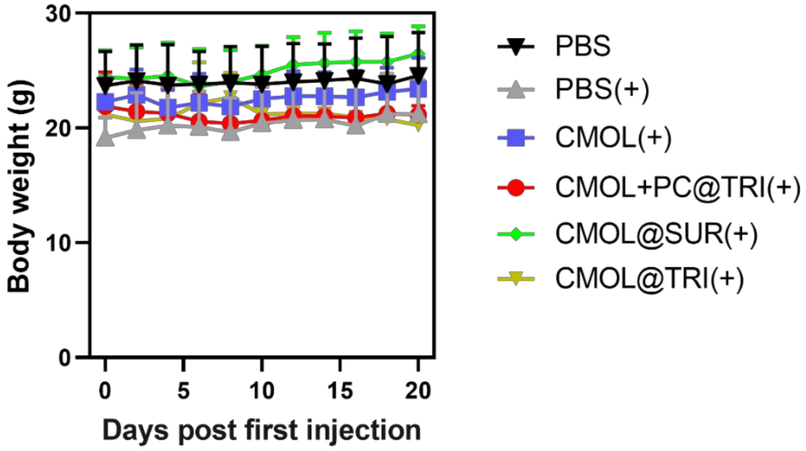

Figure S35. Body weights of subcutaneous 4T1-tumor-bearing BALB/c mice after different treatments (n=5).

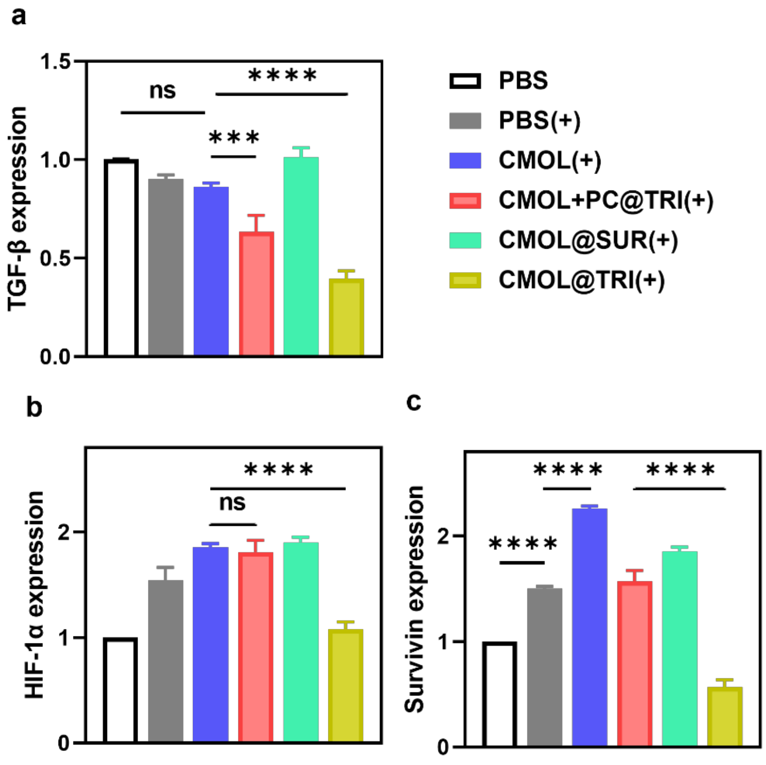

Figure S36. Relative expression analysis of in vivo western blots of (a) TGF-β, (b) HIF-1α and (c) survivin expressions in 4T1 tumors after treatment with PBS, PBS(+), CMOL(+), CMOL+PC@TRI(+), CMOL@SUR(+), or CMOL@TRI(+).

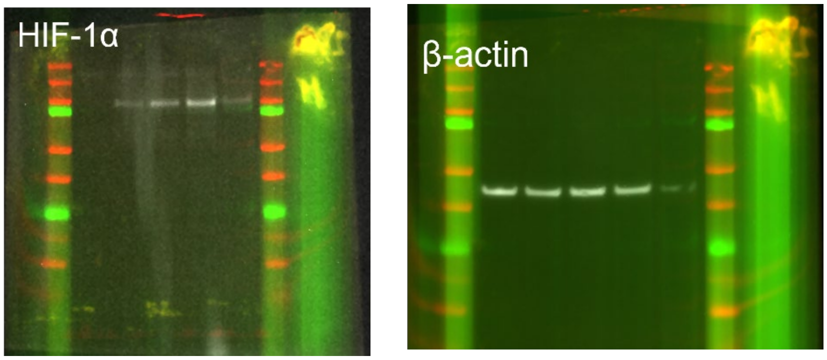

Figure S37. Uncropped blot for Figure S9. Expression of HIF-1α (with β-actin as the reference).

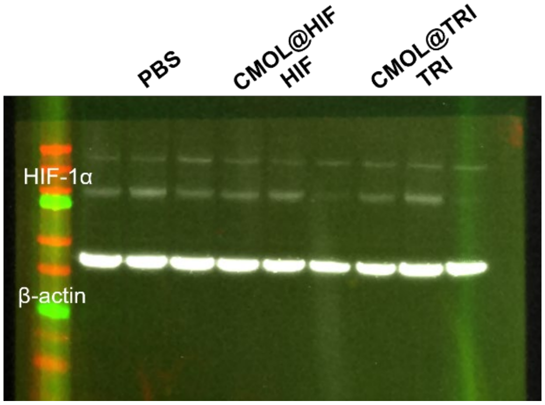

Figure S38. Uncropped blot for Figure S10. Expression of HIF-1α (with β-actin as the reference).

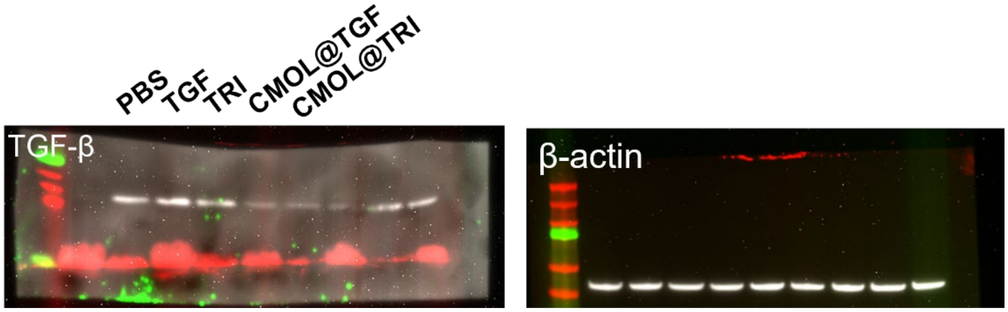

Figure S39. Uncropped blot for Figure S11. Expression of TGF-β (with β-actin as the reference).

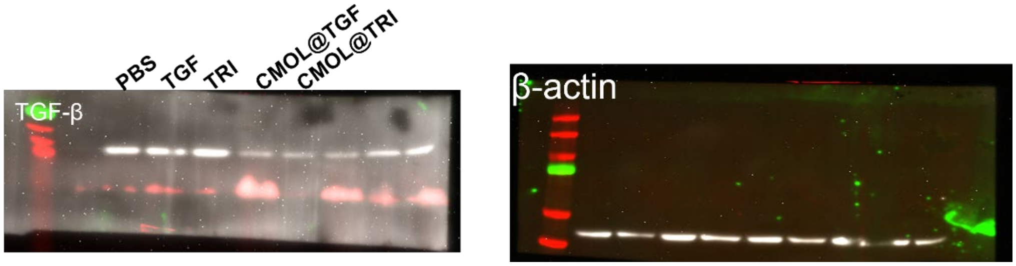

Figure S40. Uncropped blot for Figure S12. Expression of TGF-β (with β-actin as the reference).

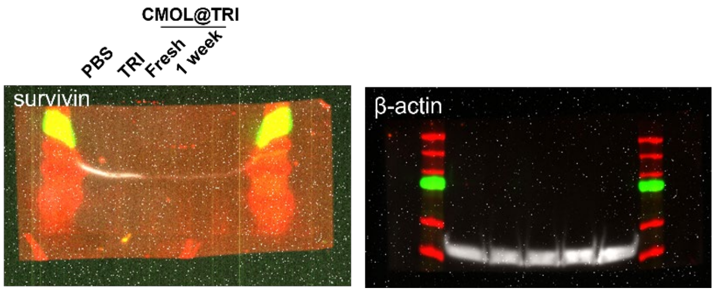

Figure S41. Uncropped blot for Figure S13. Expression of survivin (with β-actin as the reference).

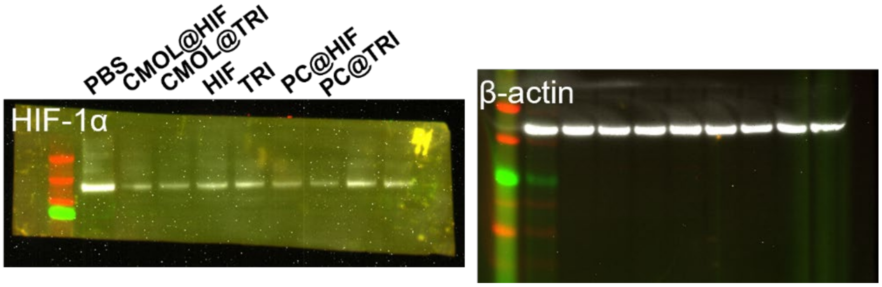

Figure S42. Uncropped blot for Figure 3b. Expression of HIF-1α (with β-actin as the reference).

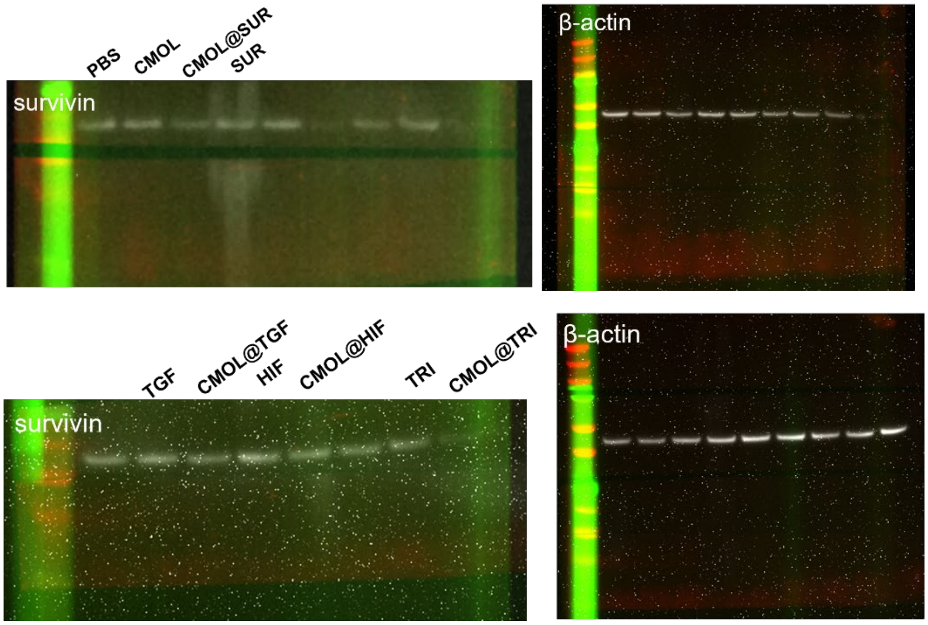

Figure S43. Uncropped blot for Figure 3d. Expression of survivin (with β-actin as the reference).

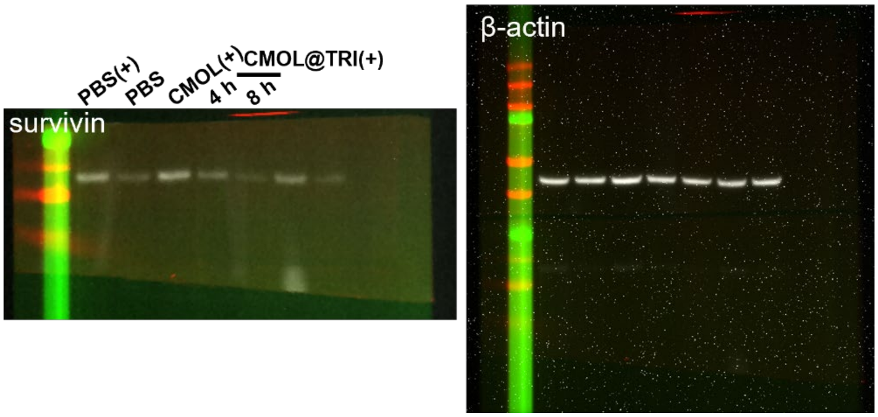

Figure S44. Uncropped blot for Figure 4e. Expression of survivin (with β-actin as the reference).

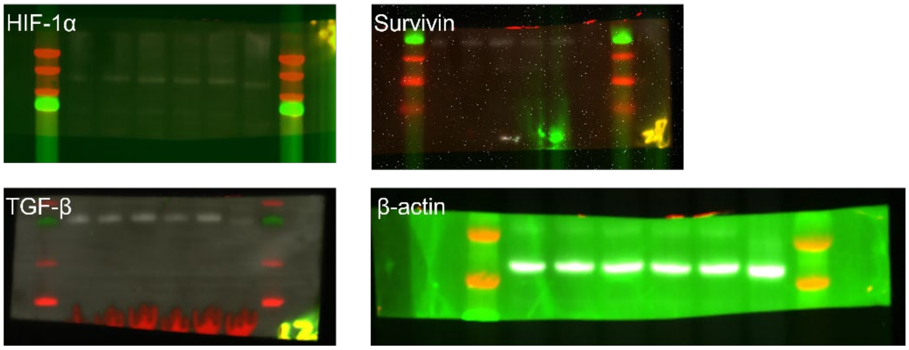

Figure S45. Uncropped blot for Figure 5e. Expression of survivin (with β-actin as the reference).

Table S1 loading efficiency of siRNA on CMOL.

	**CMOL@siRNA (wt ratio)**		
	1:1	5:1	10:1
siRNA loading efficiency	66%	100%	100%

Table S2. TGI values of CT26-bearing BABL/c mice at day 21.

**Treatment**	**TGI (CT26)**
PBS(+)	0.543
CMOL(+)	0.819
TRI(+)	0.854
CMOL+PC@TRI(+)	0.813
CMOL@HIF(+)	0.921
CMOL@TGF(+)	0.924
CMOL@SUR(+)	0.931
CMOL@TRI(+)	0.969

Table S3. TGI values of 4T1-bearing BABL/c mice at day 27.

**Treatment**	**TGI (4T1)**
PBS(+)	0.388
CMOL(+)	0.535
CMOL+PC@TRI(+)	0.502
CMOL@SUR(+)	0.582
CMOL@TRI(+)	0.914

References

[1] P. Bankhead, M. B. Loughrey, J. A. Fernández, Y. Dombrowski, D. G. McArt, P. D. Dunne, S. McQuaid, R. T. Gray, L. J. Murray, H. G. Coleman, J. A. James, M. Salto-Tellez, P. W. Hamilton, M. B. Loughrey, J. A. Fernández, Y. Dombrowski, D. G. McArt, P. D. Dunne, S. McQuaid, R. T. Gray, L. J. Murray, H. G. Coleman, J. A. James, M. Salto-Tellez, P. W. Hamilton, *Sci. Rep.* **2017**, *7*, 16878.
[2] K. Lu, C. He, W. Lin, *J. Am. Chem. Soc.* **2014**, *136*, 16712–16715.
[3] K. Lu, C. He, N. Guo, C. Chan, K. Ni, G. Lan, H. Tang, C. Pelizzari, Y.-X. Fu, M. T. Spiotto, R. R. Weichselbaum, W. Lin, *Nat. Biomed. Eng.* **2018**, *2*, 600–610.
